# A quantitative high-throughput screening pipeline to identify small molecule inhibitors of Chikungunya nsP2 protease

**DOI:** 10.1038/s41598-025-14697-3

**Published:** 2025-09-29

**Authors:** Shuaizhang Li, Xin Hu, Yong-Mo Ahn, Angelica Medina, Lin Ye, Audrey Heffner, Simon Messing, John-Paul Denson, Dominic Esposito, Emily M. Lee, Natalia J. Martinez

**Affiliations:** 1 National Center for Advancing Translational Sciences, National Institutes of Health, Rockville, MD USA; 2 Protein Expression Laboratory, Cancer Research Technology Program, Frederick National Laboratory for Cancer Research, Frederick, MD USA

**Keywords:** Chikungunya virus, CHIKV, qHTS, Drug repurposing, nsP2 protease, Drug screening, Proteases, Screening, Small molecules

## Abstract

Chikungunya virus (CHIKV) is a mosquito-borne RNA virus that has emerged as one of the most important global arboviral threats in the last decade. Although the first CHIKV vaccine has recently been FDA approved for use in healthy adults at increased risk, to date, there are no available antiviral drugs for CHIKV infection. CHIKV nsP2 protease plays a crucial role in the processing of the viral polypeptide precursor to release enzymes required for viral replication, thus making it a promising drug target for antiviral discovery. Here, we established a high-throughput pipeline to identify small molecule inhibitors of nsP2 proteolytic activity. The pipeline is composed of a suite of 1,536-well in vitro assays to support quantitative high-throughput (qHTS) screening campaigns. Specifically, we developed a fluorescence resonance energy transfer (FRET)-based assay using a fluorogenic peptide substrate encompassing an endogenous cleavage site and purified recombinant protease domain (nsP2pro). Using this assay, we interrogated ~ 31,000 unique small molecules, including those in drug repurposing libraries as well as chemically diverse and medicinal chemistry-friendly compounds. Hits were selected for follow-up validation against full-length nsP2 and an additional peptide. FRET-based 1,536-well assays for Papain, hepatitis C virus NS3-4A, and human Furin proteases were implemented to characterize compound selectivity. Notably, we developed a high-throughput cell-based proteolytic assay using a split nanoluciferase reporter to identify cell-active hits. Novel compounds were found to be potential nsP2 inhibitors and molecular docking analyses were performed to explain the binding mode of selected hits. In vitro antiviral activity was evaluated for a subset of compounds using a high-throughput CHIKV infection assay. To our knowledge, the pipeline presented here is unprecedented for CHIKV antiviral discovery research.

## Introduction

Chikungunya virus (CHIKV) is an Old-World alphavirus belonging to the Togaviridae family and is primarily transmitted to humans by *Aedes* species mosquitoes. CHIKV infection leads to an illness characterized by incapacitating fever, arthritis, and muscle pain, for which there is no effective antiviral drug treatment to date^1^. Although historically considered a tropical pathogen, adaptation of the virus to mosquito species common in temperate zones, global climate change, as well as other factors, has resulted in significant disease spread across the world^2^. CHIKV is considered a Category B priority pathogen by the National Institute of Allergy and Infectious Diseases^3^. CHIKV’s threat to public health argues for development of effective therapeutics suitable for outbreak containment. Recently, the first CHIKV vaccine was approved in the United States and European Union for use in adults at risk of infection, however, it is not currently approved for individuals under 18 years old or those immunocompromised. In addition to the uncertainty in vaccine adherence of affected countries, long-term antibody persistence is still being evaluated^4^. Altogether, an effective post-infection antiviral is needed.

CHIKV has a 11.8 kb positive-sense RNA genome that contains two open reading frames encoding at least four nonstructural and five structural proteins, respectively^5^. The nonstructural polyprotein precursor is translated first by the host machinery and is subsequently cleaved in *cis* and *trans* into the individual and functionally distinct mature proteins nsP1, nsP2, nsP3, and nsP4 by the virus-encoded nsP2 protease^5–7^. Given that nsP2-mediated proteolytic cleavage of viral precursors is critical for viral replication^8^, nsP2 constitutes a potential antiviral drug target.

The protease domain of nsP2 (referred to as nsP2pro) maps to the C-terminal region of full-length nsP2 and its active site consists of a Cys/His catalytic dyad that resembles Papain-like cysteine proteases^9^. Although initial mutational studies suggested that the Cys residue in the dyad is catalytically interchangeable with a nearby Ser residue not present in the nsP2pro of other alphaviruses^10^, later studies contradicted the role of the Ser residue and it was concluded that CHIKV nsP2 is a classical alphavirus protease^8^. In addition to its protease activity, full-length nsP2 also displays nucleoside triphosphatase (NTPase), helicase, and RNA-dependent 5′-triphosphatase activities mapping to the N-terminal half of the protein, and a SAM-dependent RNA methyltransferase-like (SAM MTase-like) domain at the very C-terminal part^11,12^. Both full-length and truncated nsP2pro domain can proteolytically cleave small peptides substrates encompassing the natural polyprotein cleavage sites, with longer peptides being cleaved more efficiently than shorter ones^8,13–15^. Substrate specificity studies indicated that while nsP2pro has a preferential affinity for the nsp3/4 over nsp1/2 and nsp2/3 sites, full-length nsP2 can efficiently cleave peptides spanning all three cleavage sites^13^.

Although previous studies have sought to discover nsP2pro inhibitors, including structure-based computer-aided drug design and low-throughput FRET-based nsP2pro assays, no potent and selective compounds have been identified for inhibition of CHIKV nsP2pro^16–21^. Here, we developed a suite of 1,536-well format assays for the identification of nsP2pro small molecule inhibitors using quantitative high-throughput (qHTS). qHTS paradigms have lower false positive and false negative rates, allow the generation of concentration–response curves (CRCs) directly from the primary screen, and both potency and efficacy values can be used to identify compounds with robust bioactivity profiles^22,23^. Specifically, we developed a FRET-based proteolytic assay that utilizes a 15-amino acid peptide substrate, which significantly speeds up reaction time. Using this assay, we screened both a drug repurposing collection of ~ 9 K compounds as well as ~ 25 K medicinal chemistry-friendly compounds. Subsequently, active compounds were validated against full-length nsP2 as well as an additional peptide containing the ns2/3 cleavage site. To evaluate compound specificity, hits were cross-interrogated in assays containing either Papain, a cysteine protease from *Carica papya*, the hepatitis C virus (HCV) NS3-4A serine protease, or human Furin, a subtilisin-like serine protease. Our pipeline incorporates a novel cell-based proteolytic assay that uses a split nanoluciferase reporter to identify cell acting hits. We report the identification of small molecules with nsP2pro inhibitory activity. Altogether, these compounds not only constitute potential new starting points for lead optimization of CHIKV nsP2pro inhibitors, but they may also represent opportunities for repurposing as well as contribute to the design of synergistic drug combinations against CHIKV. We disclose an unprecedented pipeline for CHIKV antiviral discovery applicable to the screening of any large compound library.

## Results

### Biochemical CHIKV nsP2pro assay development and optimization

**Fig. 1:**
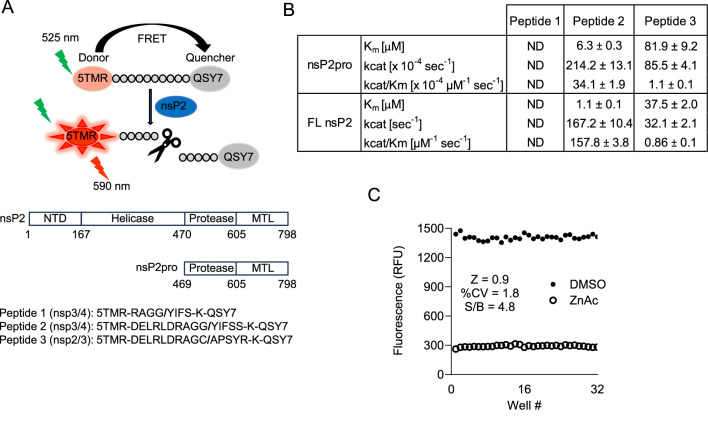
FRET-based nsP2pro assay development and optimization. (**A**) Top: Schematic representation of the fluorogenic CHIKV nsP2pro assay. Intact substrates exhibit low fluorescent emission upon excitation of 5TMR fluorophore (donor) due to quenching by proximal QHY7 (acceptor). Substrate cleavage mediated by nsP2 protease breaks the proximity between fluorophore and quencher resulting in increased fluorescence signal. Middle: schematic representation of modular organization of full length^12^ and truncated nsP2 proteases utilized in this study. NTD: N-terminal domain, MTL: SAM MTase-like domain. Bottom: substrate sequences utilized in this study. Slash symbol indicates cleavage site. (**B**) Enzyme kinetics determined using peptides 1, 2, and 3 and nsp2pro and nsp2 full-length enzymes. ND = not detected. Data represents Average + /- standard deviation (n = 3). (**C**) Scatter plot of ZnAc or DMSO signal values in 1536-well format. Values were used to calculate assay statistical parameters of Z factor (Z), percent Coefficient of Variation (%CV) and Signal-to-Background (S/B).

To accelerate the discovery of antiviral small molecules against CHIKV nsP2, we sought to develop a high-throughput assay using recombinant nsP2pro. To this end, we expressed and purified the C-terminal 329 aa of CHIKV nsP2, herein referred to as nsP2pro (Fig. 1A, Sup. Fig. 1A). A previous study demonstrated that nsP2pro cleaves a self-quenching fluorogenic peptide, separating fluorophore from quencher and resulting in increased fluorescence signal^24^. Specifically, authors used an octapeptide containing the enzyme’s natural nsp3/4 cleavage site, RAGG/YIFS, fused to the EDANS fluorophore and DABCYL quencher. Although the assay showed robust parameters, the cleavage reaction was incubated for 6 hr^24^, which is not ideal for large HTS campaigns. First, we adopted a similar peptide design but since compound-mediated fluorescence interference is prominent in the blue-green spectra of the EDANS fluorophore^25^, we red-shifted the assay by utilizing a 5-TAMRA and QSY7 fluorophore/quencher pair (peptide 1, RAGG/YIFS; Fig. 1A). However, peptide 1 was not efficiently cleaved by nsP2pro as the increase in fluorescence signal was less than two-fold compared to background, even after a 16 h incubation (Sup. Fig. 2A). We next re-designed the substrate by increasing the peptide length to span 10 amino acids before and 5 amino acids after the scissile bond of the nsp3/4 boundary (peptide 2, DELRLDRAGG/YIFSS; Fig. 1A). Peptide 2 was efficiently and rapidly cleaved by nsP2pro and was used to calculate steady-state kinetic parameters (Fig. 1B, Sup. 2B,C). To further optimize assay conditions using peptide 2, we titrated the enzyme concentration at a fixed substrate concentration of 5 µM. Enzyme concentration above 150 nM provided S/B over threefold (Sup. Fig. 2D, E). To evaluate if nsP2pro cleaves additional substrates, we tested an equal length peptide spanning the nsp2/3 cleavage site (peptide 3, DELRLDRAGC/APSYR; Fig. 1A). Although nsP2pro was able to cleave this peptide, the reaction was slower, and the catalytic efficiency was lower compared to the nsp3/4 site (Fig. 1B and Sup. Fig. 3A,B). Moreover, a higher enzyme concentration of at least 5 µM was required to obtain an assay window over threefold (Sup. Fig. 3C,D). This agrees with previous studies indicating that nsP2pro has a higher affinity for the nsp3/4 versus nsp1/2 and nsp2/3 cleavage sites^13^. Finally, we tested the assay sensitivity to vehicle DMSO and found that DMSO has minimal effect on assay signal even at concentrations as high as 10% (Sup. Fig. 2F).

To expand on the therapeutic potential of putative inhibitors, we expressed and purified full-length nsp2 (Fig. 1A, Sup. Fig.1B). Optimization of reaction conditions indicated that full-length protein had a higher catalytic efficiency for peptide 2 compared to the truncated protease domain (Fig. 1B and Sup. Fig. 4A,B) and enzyme concentrations above 80 nM provided S/B over threefold (Sup. Fig. 4C,D). Like the truncated protease domain, the affinity of full-length enzyme was higher for peptide 2 than peptide 3, which contrasts previous studies indicating equal affinity^13^ (Fig. 1B and Sup. Fig. 5A,B). We validated the assays against the previously identified inhibitor ZnAc^24^, which inhibited the nsp3/4 cleavage with an IC_50_ of 192 ± 12 nM and 137 ± 11 nM for nsP2pro and full-length enzymes, respectively. ZnAc inhibited the cleavage of nsp2/3 peptide by nsp2pro with an IC_50_ of 685 ± 77 nM (Sup. Fig. 6).

### Drug repurposing screen for nsP2pro inhibitors

Given the higher yield of recombinant protein, we selected 150 nM nsP2pro enzyme and 2.5 µM peptide 2 substrate concentrations as an optimized condition for high-throughput screening. The assay was miniaturized to 1,536-well format to support qHTS and using ZnAc as control, the assay parameters indicated a robust performance suitable for HTS (Fig. 1C). To identify new therapeutic options for CHIKV treatment, we interrogated the assay against four drug repurposing small molecule libraries sourced by the National Center for Advancing Translational Sciences (NCATS): the NCATS Protease Inhibitor Collection (NPIC), the NCATS Pharmaceutical Collection (NPC), the NCATS Pharmacologically Active Chemical Toolbox (NPACT), and the NCATS anti-infectives collection. Altogether, the screen comprised 9,937 approved and investigational drugs as well as highly annotated tool compounds with varying degrees of validation as therapeutics^26–28^. This primary screen identified 253 unique compounds (3% hit rate) with nsP2pro inhibitory activity defined as high quality concentration response curve (CRC), efficacy > 50%, and IC_50_ < 10 μM (described in Materials and Methods) and of these, 250 available compounds were sourced for follow-up validation studies in the same enzyme assay. Hit validation was performed at 11-point concentrations and confirmed the activity of 116 unique compounds (46% validation rate).

**Fig. 2:**
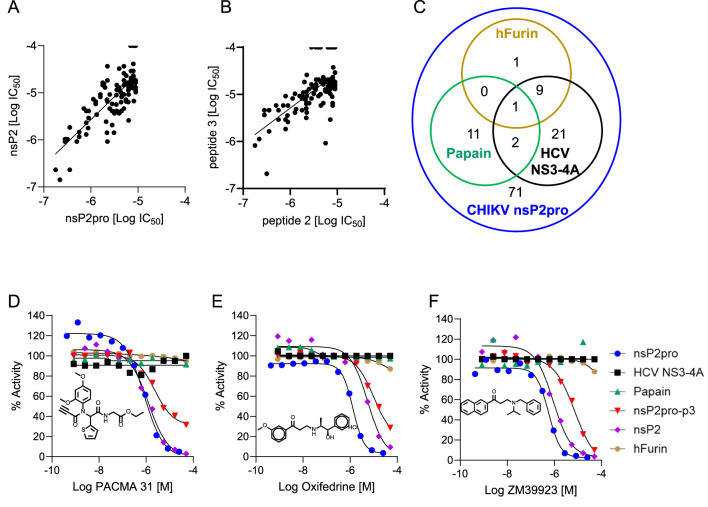
Assay screening of repurposing libraries. (**A**) Correlation plot of compound inhibitory activity (Log IC_50_) in truncated protease domain vs. full-length nsP2 enzymatic reactions (R^2^ = 0.60). (**B**) Correlation plot of compound inhibitory activity (Log IC_50_) of nsP2pro with peptide 2 vs. peptide 3 substrates (R^2^ = 0.43). (**C**) Selectivity characterization of hits. Venn diagram indicating number of compounds with activity in Papain, Furin, NS3-4A, and nsP2pro assays. (**D**–**F**) Example of dose response curves for selective hits identified in follow-up and counterscreen assays: nsP2pro (blue), nsP2pro-peptide 3 (red), nsP2 full-length (purple), Papain (green), hFurin (brown), and HCV NS3-4A (black).

To gain further insight on their therapeutic potential, we investigated whether confirmed hits inhibit proteolytic activity in the context of full-length nsP2 protein rather than isolated protease domain^13^. To test the set of confirmed inhibitors, we chose peptide 2 substrate and full-length enzyme concentrations of 1 μM and 150 nM, respectively. Compound activity correlated well between nsP2pro and full-length enzyme (Fig. 2A). Additionally, we tested whether hits inhibit proteolysis of the nsP2/3 site. Using peptide 3 as substrate and nsP2pro at concentrations of 10 and 1 µM, respectively, most compounds also inhibited proteolytic activity, albeit the overall inhibitory potency was lower compared to peptide 2 (Fig. 2B).

To characterize compound selectivity, we screened validated hits for activity against Papain, HCV NS3-4A, and Furin proteases, as previously described^29–31^. All three proteolytic FRET-based assays were run in 1536-well format. While Papain and Furin assays utilize a 7-amino-4-methylcoumarin (AMC)-based fluorogenic peptide fluorescent on the green spectra, the HCV NS3-4A assay utilizes a peptide with red-shifted TAMRA fluorophore. These counterscreens identified 45 compounds with activity (curve class − 1 and − 2, > 50% efficacy) against one or more other proteases besides nsP2pro, while the remaining 71 compounds displayed preferential activity for nsP2pro (Fig. 2C). Examples of selective hits include the protein disulfide isomerase (PDI) inhibitor PACMA 31, the adrenoreceptor agonist Oxyfedrine, and the Janus tyrosine kinase (Jak) 1/3 inhibitor ZM39923 (Fig. 2D,F). We found that similar to ZnAc, Zinc dibutyldithiocarbamate was a potent inhibitor of the cysteine proteases tested but was either weak or had no effect on serine proteases (Sup. Figure 7A). Interestingly, our screen did not identify cysteine protease inhibitors such as the fluoromethyl ketones Z-FA-FMK and Z-DEVD-FMK. These compounds have been shown to inhibit other viral proteases such as SARS-CoV2 3CL but not PLpro^27,32^. Accordingly, Z-FA-FMK had minimal inhibitory activity against CHKV nsP2 while potently inhibiting Papain (Sup. Figure 7B). Supporting our findings, docking Z-FA-FMK into the substrate binding sites indicated that it has full access to the substrate pocket in Papain, but access is blocked by the MTase domain or the flexible D-loop in CHIKV-nsP2 and SARS-CoV-2 PLpro, respectively (Sup. Figure 8A-C). Similarly, HCV protease inhibitors were not identified in the screen. Telaprevir, while potently inhibiting NS3-4A and Papain, had potencies above the 10 µM cutoff we applied to the CHIKV nsP2 screen (Sup Fig. 7C). These results indicate our screening pipeline identifies protease inhibitors, some with preferential nsP2 inhibition with potencies below 10 µM.

### Identification of nsP2pro inhibitors from chemically diverse libraries

**Fig. 3:**
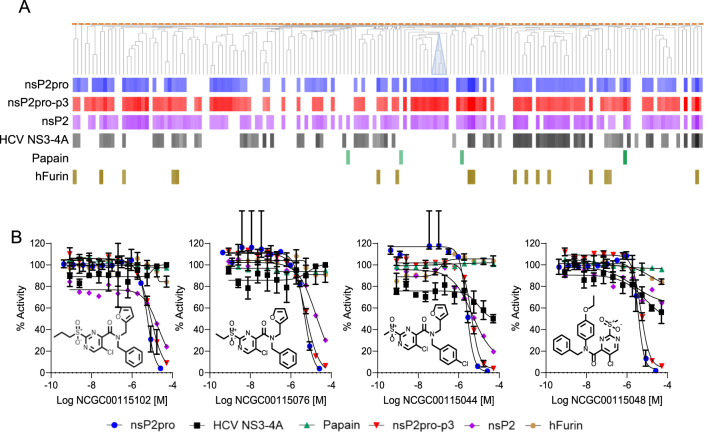
Assay screening of diversity libraries. (**A**) Structure-based clustering of the 116 hits from diversity libraries sourced for validation. Activity outcome in each enzymatic assay is shown as a potency (LogIC_50_) gradient with dark colors indicating high potency and light colors, low potency. White indicates lack of activity. Blue triangle shows the nsP2pro-selective sulfonylpyrimidine scaffold. (**B**) Dose–response curves of 4 analogs containing the sulfonylpyrimidine scaffold (Tanimoto > 0.9) in all enzymatic assays. nsP2pro (blue), nsP2pro-peptide 3 (red), nsP2 full-length (purple), Papain (green), hFurin (brown), and HCV NS3-4A (black).

To identify novel chemotypes that could serve as starting points for medicinal chemistry development, we screened a collection of 25,994 NCATS-sourced chemically diverse small molecules utilizing the pipeline of assays described above. The primary screen identified 290 compounds (1% hit rate) with nsP2pro inhibitory activity defined as high quality concentration response curve (CRC), efficacy > 50%, and IC_50_ < 10 μM, and of these, 164 available compounds were sourced for follow-up validation studies. Structure similarity analysis of these 164 compounds indicated broad ligand diversity with several clusters of 3 or more compounds containing common scaffolds (Fig. 3A). Follow-up studies confirmed the activity of 80 hits (71% hit rate). Compounds were also characterized in assays using full-length nsP2 or peptide 3 (Fig. 3A). The inhibitory activity of hits correlated well between peptide 2 and peptide 3 assays (Sup. Figure 9A). However, the correlation of compound activity between full-length and truncated nsP2 proteases was poor (Sup. Figure 9B). Selectivity characterization against Papain, HCV NS3-4A, and Furin proteases identified prominent compound activity in the HCV NS3-4 assay but minimal activity against the other two proteases (Fig. 3A and Sup. Figure 9C). Among nsP2pro-selective compounds, for example, we found a sulfonylpyrimidine scaffold was shared among 4 structurally similar analogs (Tanimoto > 0.9) that displayed inhibitory activity against nsP2pro (Fig. 3A,B).

### Cell-based proteolytic assay for characterization of nsP2 inhibitors

**Fig. 4:**
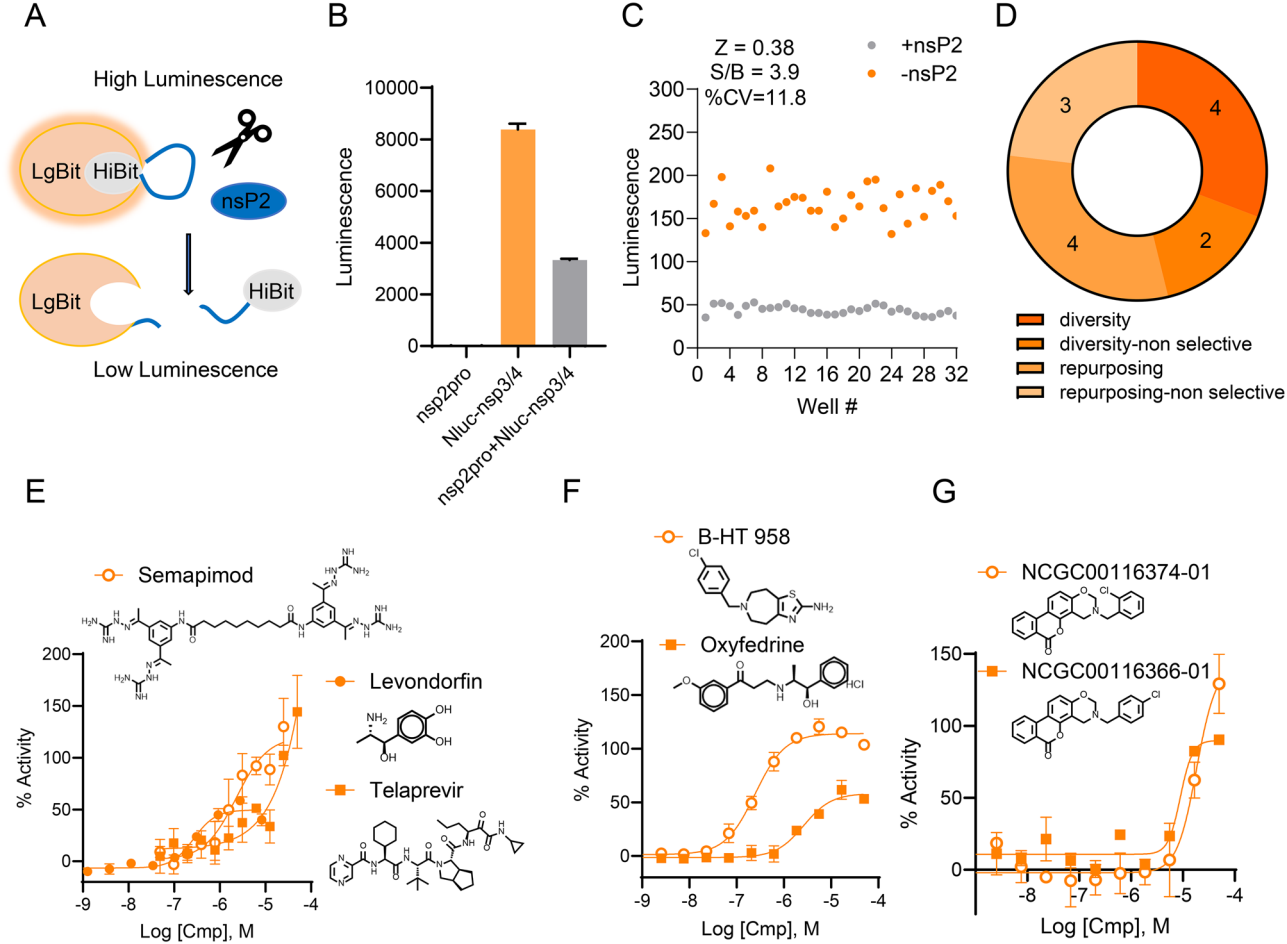
Cell-based proteolytic assay development and optimization. (**A**) Schematic representation of assay principle. Expression of split Nluc reporter system containing the CHIKV nsP3/4 cleavage site (Nluc-nsp3/4) between the large (LgBiT) and small (HiBiT) Nluc fragments produces high luminescence signal. Co-expression of an nsP2-expressing construct (nsP2pro) leads to cleavage of linker between LgBiT and HiBiT, resulting in low luminescence signal. (B) Quantification of luminescence in HEK293 cells transfected with nsP2pro construct, Nluc-nsp3/4 construct, or both. Data represents Average + /− standard deviation, n = 3. (**C**) Assay miniaturization to 1,536-well. Scatter plot of luminescence values of control wells used to calculate assay statistical parameters of Z factor (Z), percent Coefficient of Variation (%CV) and Signal-to-Background (S/B). High-signal values are obtained from cells transfected with Nluc-nsp3/4 only (-nsP2pro) and low-signal values, from co-transfection of Nluc-nsp3/4 and nsP2pro (+ nsP2pro). (**D**) Number of cell-based active compounds from repurposing and chemically diverse (diversity) libraries. Non-selective hits respect to Papain, hFurin, and HCV NS3-4A proteases are indicated. Dose–response curves in cellular protease assay: non-selective hits Semapimod, Levondorfin, and Telaprevir (**E**); adrenergic agonists B-HT 958 and Oxyfedrine from repurposing libraries; and (**G**) structurally similar hits from chemically diverse libraries. Data represents Average + /− standard deviation, n = 3.

We next sought to develop a cell-based assay amenable for high-throughput screening that would allow the triage of cytotoxic as well as cell-inactive compounds such as those with poor permeability. A cell-based enzyme complementation assay has been described for SARS-CoV2 3C-like protease (3CLpro)^33^. The assay principle involves a lentiviral-based split nanoluciferase (Nluc) reporter^34^ in which the large (LgBit) and small (HiBiT) fragments of Nluc are linked by a flexible loop containing a protease cleavage site. Upon expression of the protease and cleavage of the flexible loop, both luciferase fragments are separated resulting in a decrease in luminescence, which can be reverted by inhibition of protease activity. Thus, protease inhibitors increase Nluc signal while cell-inactive or cytotoxic compounds lead to either no change or decrease in reporter signal. Artifactual Nluc reporter inhibitors would also decrease Nluc signal (Sup. Figure 10A-B). We adopted a similar strategy by engineering a plasmid-based split Nluc reporter system containing the CHIKV nsP3/4 cleavage site (Fig. 4A). Transfection of HEK293 cells with the Nluc reporter resulted in high luminescence signal, which decreased ~ 2.5 fold upon co-transfection with a plasmid expressing the nsP2 protease (Fig. 4B). nsP2 protease did not efficiently cleave an Nluc reporter system containing the ns2b/ns3 cleavage site of West Nile Virus (WNV)^35^, a positive-sense RNA virus belonging to the family Flaviviridae, or an Nluc reporter containing a 12xGlySer linker with no proteolytic site (Sup. Figure 10C,D). In contrast to 3CLpro, no potent cell-active inhibitor has previously been reported for CHIKV nsp2 that could be used to benchmark the assay. ZnAc was found effective in preventing CHIKV viral infection at concentrations higher than 60 µM^24^, which is above the top concentration tested in our miniaturized assays (49 µM). Additionally, ZnAc effect on CHIKV infection was not formally shown to be due to on-target inhibition of nsP2. Accordingly, ZnAc did not increase Nluc reporter signal in our assay (Sup. Figure 10E). We then implemented Nluc reporter signal in the presence (low signal) or absence of nsP2 (high signal) as assay controls. Although assay parameters indicated a S/B of 3.8 and Z’ of 0.38, we deemed it acceptable for a transfection-based cell assay (Fig. 4C). We tested a collection of 415 compounds including the 251 and 164 compounds sourced from repurposing and diversity libraries, respectively. While most had no effect (defined as curve class 4 or inactive), 131 compounds decreased Nluc reporter signal (curve class − 1 or − 2), as evidenced by their loss-of-function dose response curves, indicating potential cytotoxicity or luciferase inhibition. Thirteen compounds from repurposing and chemically diverse libraries that showed nsp2 proteolytic inhibition in biochemical assays increased Nluc reporter signal (high quality curve class 1 or 2 and efficacy > 50%), indicating potential inhibition of nsP2pro in the cellular context (Fig. 4D). Of these, Telaprevir, the adrenergic receptor agonist Levonordefrin, and the anti-inflammatory compound Semapimod (Fig. 4E) also inhibited the HCV NS3-4A serine protease, but not Papain. Semapimod also had weak activity against Furin. Interestingly, Levonordefrin has been proposed to target aspartic proteases of filarial worms and Semapimod was identified as a SARS-CoV2 PLpro inhibitor^36,37^. The remaining compounds were specific to nsP2pro based on biochemical assays and include the adrenoreceptor agonists Oxyfedrine and B-HT 958 (Fig. 4F) and two related analogs, NCGC00115102 and NCGC00116374, from the chemically diverse libraries (Fig. 4G). Overall, no obvious potency correlation was observed between enzymatic and cell-based assays for these 13 compounds (Sup. Figure 10F).

### Modeling hit binding to nsP2pro

**Fig. 5:**
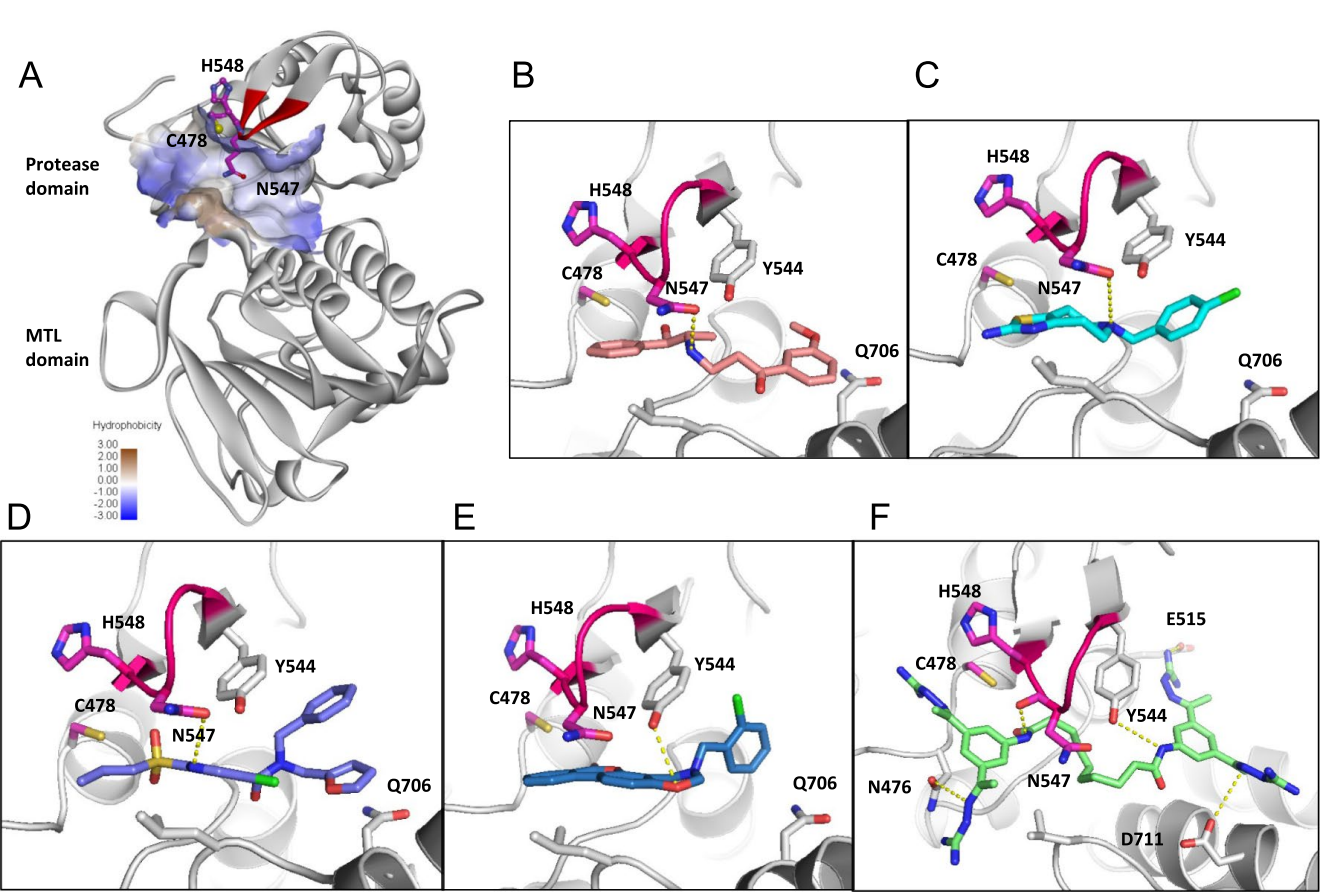
Predicted binding models of selected inhibitors to the substrate binding site of nsP2pro. (**A**) Structure of the truncated nsP2 protein containing the protease and Methyltransferase-like domains in ribbon representation. Surface representation of catalytic pocket is shown (hydrophilic in blue color, hydrophobic in white/yellow) with the catalytic dyad in sticks (pink/red). (**B**–**F**) Detailed binding models of docked inhibitors into the catalytic pocket. H-bonding interaction of key residues with inhibitors are shown in yellow. Small molecules are shown in sticks: (**B**) Oxyfedrine, (**C**) B-HT 958, (**D**) NCGC00115102, (**E**) NCGC00116374, and (**F**) Semapimod.

To further investigate potential compound binding to CHKV nsp2, we docked selected hits to the protease active site. A large substrate binding pocket of nsP2pro is formed at the interface of protease subdomain and MTase subdomain, which contains a catalytic dyad of C478/H548 and a flexible DL loop with residue N547 that points toward the active site (Fig. 5A). The DL loop is known to adopt an open and closed conformation and play an important role in substrate as well as inhibitor binding^9^. Docking studies of Oxyfedrine and B-HT 958 showed that both compounds fit well in the active site near the catalytic dyad and formed a H-bonding interaction with N547 (Fig. 5B,C). The novel series of identified inhibitors NCGC00115102 and NCGC00116374 were also accomodated well into the pocket in a similar binding manner (Fig. 5D,E). Remarkably, Semapimod bound in the substrate binding pocket by forming extensive interactions with the DL loop as well as the MTase subdomain (Fig. 5F).

### Evaluation of selected compounds in in vitro CHIKV181/25 infection assay

**Fig. 6:**
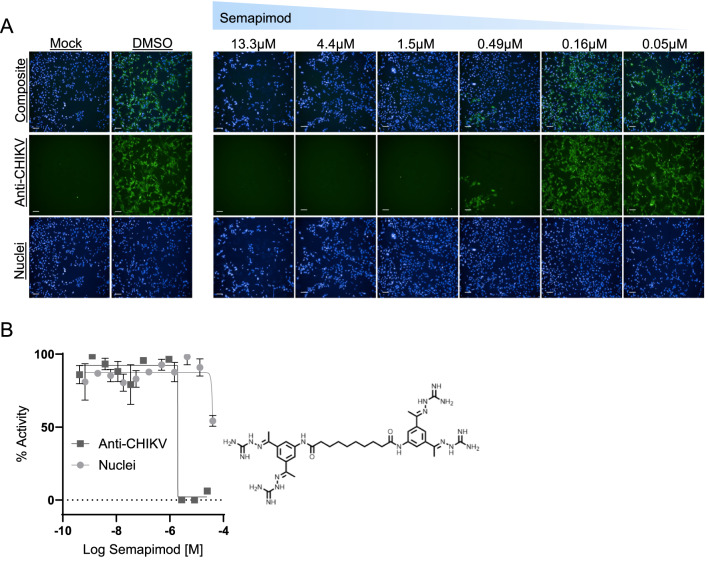
Semapimod inhibits CHIKV infection in SNB-19 cells. (**A**) Left: representative immunofluorescence images of SNN-19 cells uninfected (Mock) or infected with CHIKV and treated with vehicle DMSO. Right: representative immunofluorescence images of CHIKV infection inhibited by a dose treatment of Semapimod in SNB-19 cells. Cell nuclei are stained with Hoechst 33342 (blue channel) and virus antigen detected with anti-CHIKV antibody recognizing CHIKV E1 protein (green channel). (**B**) Dose response curve for Semapimod on inhibition of infection (green channel) and nuclei count (blue channel). Data represents average + /− standard deviation, n = 3.

As part of complementary efforts in identifying CHIKV antiviral opportunities, we have developed a 1,536-well, high content phenotypic screening assay in a permissive human cell line (Medina et al., in preparation). Briefly, cell lines susceptible to CHIKV infection were evaluated and selected for desirable characteristics needed for qHTS, including monolayer adherence in a miniaturized 1,536-well setting, virus disease relevance, and low variability on virus infection with the optimal multiplicity of infection (MOI) resulting in consistent Z’ scores > 0.6. Specifically, SNB-19 glioma cells and the CHIKV 181/25 vaccine strain were selected for the assay. For high-content imaging, virus was detected through immunofluorescence using an antibody against the CHIKV envelope glycoprotein E1 protein and imaging analysis was used to evaluate total nuclei and the percentage of infected cells in the cell monolayer. Inhibition of the infection percentage by different compounds was used as our activity readout. Nuclei count was used as a measure of compound-mediated cell toxicity. SNB-19 cells were exposed to compound treatment at 11-point concentrations and subsequently infected with CHIKV181/25 at MOI 1 and incubated for 44 h to achieve consistent infection while maintaining a cell monolayer. Gemcitabine, a cytidine analog with reported broad spectrum antiviral activity^38^ was used as our infection inhibition control compound as it showed consistent CHIKV inhibition under 1 µM concentrations (Sup. Figure 11). Since repurposing libraries have been screened using this assay (Medina et al., in preparation), we searched the activity outcome of nsP2pro hits. We first evaluated the cytotoxicity effect of selected nsP2pro hits on SNB19 cells via an independent CellTiter-Glo viability readout (Sup. Figure 10G). We found that of the 7 repurposing hits identified in the HEK293-based nsP2pro assay, NCGC0031503 partially reduced (~ 50% efficacy) SNB19 viability with a CC_50_ of 0.44 µM. Telaprevir, SPP 86 and Semapimod reduced viability only at high concentrations with CC_50_ values of 11.6, 16.4 and 20.6 µM, respectively. The remaining hits had no effect on SNB19 viability. Inspecting the effect of hits in the CHIKV viral assay, we found Semapimod strongly prevented CHIKV infection with IC_50_ of 1.6 µM, > tenfold more potent than its effect on cell viability (Fig. 6A,B and Sup. Figure 12). Interestingly, Epigallocatechin gallate (EGCG) also strongly prevented CHIKV infection (Sup. Figure 13A). However, while EGCG inhibited nsp2pro activity in vitro, it did not have an effect in the cellular nsp2 proteolytic assay, suggesting it prevents infection via an alternative mechanism. While ZnAc was not tested, the effect of high concentrations of Zn dibutyldithiocarbamate on CHIKV infection was difficult to distinguish from cell toxicity (Sup. Figure 13B).

## Discussion

Development of CHIKV antivirals constitutes, to date, an unmet need. Targeting viral proteases has been a successful strategy for antiviral development as multiple protease inhibitors have been approved for human immunodeficiency virus (HIV), HCV, and more recently, the coronavirus SARS-CoV-2^39,40^. Homology models and the crystal structure of the CHIKV nsP2 protease domain have been used to identify putative small molecule inhibitors by computer-aided drug design^16–21^. In one of these studies, compounds were shown to have antiviral activity in cellular assays, however, no experimental validation using isolated nsP2pro assays was provided to corroborate on-target inhibition^18^. Other studies utilized low-throughput cleavage assays to show compound-mediated inhibition of nsP2pro, however, the correlation of compound activity between cleavage and cellular assays was poor, indicating that antiviral activity was likely mediated in part by off-target effects^19–21^. In all cases, inhibitor potency was modest, typically in the micromolar range, and no selectivity analysis against other proteases was provided. A 96-well FRET-based protease assay was also reported to facilitate the high-throughput screening of CHIKV nsP2pro inhibitors^24^. This assay was used to screen a small panel of cations and common protease inhibitors for their effect on nsP2pro activity and found that ZnAc was a potent inhibitor. However, the reaction needed a long incubation time (~ 6 h) to achieve a good signal/background (S/B) ratio, which is not ideal when supporting high-throughput screening campaigns.

In this study, we developed a high-throughput amenable pipeline to target CHIKV nsP2 protease. Our primary FRET-based biochemical assay optimization indicated that the length of peptide substrates as well as choice of cleavage site influence the enzymatic activity of CHIKV nsP2pro. The enzyme had higher affinity for the nsp3/4 versus nsp2/3 cleavage site and preferred longer peptide substrates (15 vs. 10 amino acids) as previously reported^13^. While it has been shown that truncated nsP2pro domain does not significantly differ in proteolytic activity compared to full-length nsP2 in the context of small peptides substrates^13^, our results indicate that full-length enzyme has a higher catalytic efficiency compared to the truncated protease domain. The primary assay’s large capacity, reduced time frame, and red shift are ideal to support large scale screening campaigns. The incorporation of Papain, HCV NS3-4A, and Furin FRET-based protease assays to the screening pipeline contributes to the identification of broad-spectrum protease inhibitors and potential assay artifacts. Similarly, validation strategies assaying an additional cleavage site and full-length protein provide further insights into inhibitor’s therapeutic potential. To our knowledge, we generated the largest screening dataset against a CHIKV target to date. Furthermore, this dataset may be useful for in silico studies as it constitutes a validated training set. Inhibitors identified from diversity libraries also constitute starting points for medicinal chemistry optimization.

The inhibition in enzymatic assays cannot guarantee antiviral activity in cell-based assays for several reasons, including the compound’s cell permeability, metabolization by intracellular enzymes, active efflux by cellular pumps, and cytotoxicity. Additionally, the isolated recombinant enzymes used in biochemical assays may show different responses to inhibitors compared to the native viral enzyme in cells. To overcome some of these impediments, we developed a novel nsP2 proteolytic assay to investigate compound’s activity in cells. Our assay identified 13 hits with potential cellular activity. It is important to note that because the incubation period of cells with compounds is 24 h, we cannot rule out off-target effects leading to apparent reporter activity. When comparing compound potency between cellular and nsP2pro biochemical assays, we observe no obvious correlation. This is not surprising as disconnects between enzymatic and cellular potency are readily prevalent in early stages of drug discovery^41,42^. A recent report describing a cyclically permutated firefly luciferase reporter assay for the NS2B-NS3 protease of ZIKA virus, also observed discrepancies in compound activity between biochemical and cellular environments^43^. Finally, while this assay is currently based on transient transfection of protease and split Nluc reporter constructs, generation of stable cell lines will readily improve throughput and decrease noise to facilitate the screening of large compound collections.

Drug repurposing efforts against CHIKV infection, either with single or combination agents, have been limited in throughput^44,45^. By combining target-based CHIKV-nsP2 assays with a phenotypic IFA-based CHIKV infection assay, we were able to identify Semapimod as a potential anti-viral agent for the treatment of CHIKV infection. To our knowledge, this is a novel finding since Semapimod has not been previously reported to inhibit CHIKV infection, either as single or combination agent. As Semapimod is a multifunctional compound^46,47^, the mediation of its antiviral activity through nsP2 inhibition needs to be further validated. However, reported MOAs of Semapimod as an immunomodulatory and anti-inflammatory agent may provide additional benefit to CHIKV-infected patients, where the inflammatory response driven by viral infection results in severe disease. Altogether, we propose the use of Semapimod as potential therapeutic should be investigated further. Compounds that were active in the cell-based proteolytic assay but did not show antiviral activity as single agents may still constitute opportunities for synergistic drug combinations, especially in combination with compounds targeting other CHIKV proteins. While evaluation in live virus assays is still pending for hits derived from diversity libraries, our pipeline identified compounds that could be expanded on through analog generation and further SAR studies to improve on cellular proteolytic activity.

## Materials and methods

### Compounds

Zinc acetate, and dimethyl sulfoxide (DMSO) were purchased from Sigma-Aldrich. All libraries were sourced from the National Center for advancing Translational Sciences (NCATS). The NCATS Protease Inhibitor Collection (NPIC) contains 943 annotated compounds. The anti-infectives library contains 739 bioactive/annotated compounds that specifically target viruses. The NPC library contains 2,807 compounds that are FDA approved or investigational drugs. The NPACT library contains 5,448 structurally diverse compounds (including approved and investigational drugs, and natural products) with known target or mechanism of action. Altogether, 9,937 compounds were tested as part of the repurposing set. The medicinal chemistry-friendly compounds included a subset of the PubChem collection, a subset of the Genesis collection, and the NCGC Chem collection, totaling 25,994 small molecules. Taking into account some compounds are present in one or more libraries, the total number of unique compounds screened was 31,413.

### Recombinant protein production

CHIKV nsP2 constructs were generated by DNA synthesis at ATUM, Inc. Amino acid sequences consisted of residues 1–798 (full-length) or 469–798 (protease domain) of the CHIKV nsP2 protein preceded by a tobacco etch virus (TEV) protease cleavage site (ENLYFQ/G), and DNA sequences were optimized for expression in *E. coli*. In the case of the full-length protein, a C798A mutation was introduced to eliminate aggregation issues as previously shown^8^. Synthetic DNAs were generated as Gateway Entry clones and subcloned using Gateway LR recombination into pDest-566 (Addgene: 11,517) to produce bacterial expression clones introducing an aminoterminal His6-MBP fusion tag in front of the nsP2 genes.

Bacterial expression constructs were transformed into *E. coli* BL21-star (DE3) [pRare] cells and plated on LB agar with 100 µg/mL ampicillin and 15 µg/ml chloramphenicol at 37 °C. For each construct, a seed culture was started from a single colony in 50 mL of MDAG-135 medium and grown overnight at 37 °C. Ten mL of seed culture was used to inoculate 500 mL of Dynamite medium in a 4L baffled shake flask. Cells were grown at 37 °C to an OD600 of 6–8, before adding 0.5 mM IPTG to induce protein expression at 16 °C for 18 h. Cells were collected by centrifugation at 4,000 × g for 10 min and frozen at -80 °C.

Cell pellets were thawed and resuspended in lysis buffer (20 mM HEPES, pH 7.4, 500 mM NaCl, 1 mM TCEP, 10% glycerol, 1:200 v:v Sigma Protease Inhibitor Cocktail #P8849) using a volume of 1 mL/100 OD600 units. Cells were lysed in a microfluidizer with 2 passes at 10,000 psi. Lysates were clarified by ultracentrifugation at 100,000 g for 30 min at 4 °C, followed by filtration of the soluble fraction with a 0.45 µm PES filter. Chromatography was conducted at room temperature (~ 22 ◦C) using NGC medium-pressure chromatography systems from BioRad Laboratories Inc. (Hercules, CA). The lysates were adjusted to 35 mM imidazole and loaded onto equilibrated IMAC (immobilized metal affinity chromatography) columns (Ni Sepharose High Performance nickel-charged resin, Cytiva, Marlborough, MA) at a ratio of 20 mL of resin/L of culture. Column equilibration buffer was 20 mM HEPES, pH 7.4, 500 mM NaCl, 1 mM TCEP, 10% glycerol plus 35 mM imidazole; the imidazole is added to reduce non-specific binding to the purification resin. The columns were washed to baseline with the equilibration buffer. A 10 column-volume (CV) gradient was implemented to 500 mM imidazole, followed by a 2 CV wash of 500 mM imidazole. SDS-PAGE and Coomassie-staining were used for elution fraction analysis. Appropriate fractions were pooled, His6-TEV protease (purified in-house) at 4 mg/mL was added at a 1:20 (v/v), and the digestion pool dialyzed using 10 K MWCO membrane (SnakeSkin™ Dialysis Tubing, Thermo Fisher Scientific) against the equilibration buffer without imidazole overnight at 4 ◦C. Another IMAC was performed using the same Ni Sepharose High Performance nickel-charged resin (Cytiva, 20 mL of resin/L of culture), no imidazole was present in the equilibration or wash buffers. A 5 CV gradient was performed to 100 mM imidazole, followed by a 2 CV wash of 500 mM imidazole. Appropriate fractions were pooled after SDS-PAGE and Coomassie-staining analysis and concentrated using 10 K MWCO (for CHIKV.nsP2pro) and 30 K MWCO (for CHIKV.nsP2) Amicon centrifugation units (EMD Millipore) to an appropriate volume for size exclusion chromatography (SEC).

The concentrated pool was loaded onto an equilibrated HiLoad 16/ 600 Superdex 75 resin (Cytiva) column in final buffer of 20 mM HEPES, pH 7.4, 500 mM NaCl, 1 mM TCEP, 10% glycerol. Appropriate fractions were pooled using SDS-PAGE and Coomassie-staining analysis and concentrated if needed by the above 10 K and 30 K MWCO Amicon centrifugation units and filtered with a Millex-GP 0.22 μM syringe filter (EMD Millipore). The protein concentration was determined at 280 nm (Nanodrop 2000C Spectrophotometer, Thermo Fisher Scientific), and samples were snap frozen in liquid nitrogen in 50 μL aliquots in 1.5 mL Eppendorf tubes. Protein samples were analyzed by electrospray ionization mass spectrometry (ESI–MS) using an atmospheric pressure ionization source coupled to an Exactive Plus EMR Orbitrap Mass Spectrometer (Thermo Fisher Scientific). High-resolution intact protein mass (MS1) spectra were acquired over a 600–2500 m/z window at 120,000 FT resolution (at 400 m/z) with an automatic gain cutoff target value of 3e + 06 and averaging 4 microscans. Protein purity was determined by densitometry of SDS-PAGE images using ImageJ.

### Biochemical assay optimization

The FRET peptides 5-TAMRA-RAGGYIFSS-K-QSY7 (peptide 1), 5-TAMRA-DELRLDRAGGYIFSS-K-QSY7 (peptide 2), and TMR-DELRLDRAGC/APSYR-K-QSY7 (peptide 3) were purchased from CPC Scientific Inc. and dissolved in DMSO. The initial assay was developed in 96-well black plates (#655076, Greiner One) in a volume of 100 µL. Different concentrations of substrate (as indicated in each figure) in assay buffer (10 mM Tris-HCI, pH 8.0, 0.01% Tween) were mixed with nsP2pro or full-length enzyme in triplicates. The enzyme concentration used was 1 µM, 200 nM, and 2 µM for peptides 1, 2, and 3, respectively. Kinetic fluorescence readings were obtained at 5 min intervals for 90 min using ViewLux plate reader (PerkinElmer, USA) at an excitation wavelength (λex) of 525 nm and an emission wavelength (λem) of 590 nm. Kinetic parameters (K_m_, V_max,_ and kcat) based on the Michaelis–Menten curve were calculated using GraphPad Prism software. Optimization of enzyme concentration was carried out as above except substrate was kept at 5 µM and enzyme concentration was varied as indicated in each figure. Reaction sensitivity to DMSO was performed as above but at fixed substrate and enzyme concentrations of 5 µM and 150 nM of peptide 2 and nsP2pro, respectively. All reactions were incubated at room temperature (RT).

Two point five μM peptide 2 substrate and 150 nM nsP2pro were selected to run the assay into 1536-well plate format. ZnAc was used as a positive control in the assay (50 µM). Briefly, 20 nL DMSO, control ZnAc, and test compounds were transferred into a 1,536-well solid bottom black plate (789176-F, Greiner One) via Echo 655 acoustic dispenser (Beckman Coulter). For primary screens, compounds were tested at 7 concentrations, 1:3 dilution points ranging from 25 µM to 34 nM. Follow-up confirmatory screens were carried out at 11 concentrations, 1:3 dilution points from 25 µM to 0.42 nM. Four µL nsP2pro enzyme mix in assay buffer (10 mM Tris⋅HCl pH 8.0 with 0.01% Tween 20) was dispensed into the plate using a BioRAPTR FRD liquid dispenser (Beckman Coulter). The plate was incubated at RT (protected from light) for 15 min before addition of 4 µL substrate in assay buffer. After 1 h, plates were immediately read on a ViewLux high-throughput CCD imager (Exposure = 10 s, Gain = High, Speed = Slow, Binning = 2X). The above assay was also incorporated in the NCATS HTS facility^48^, which allowed for robotic liquid and compound dispensing, microplate handling, and fluorescence reading. Follow-up experiments utilizing peptide 3 were carried out as above except that 10 μM substrate and 1 μM nsP2pro were utilized.

### Biochemical selectivity screens

The 1,536-well FRET assay to monitor Papain (Sigma) activity using Z-FR-AMC (Bachem) peptide substrate was performed as previously described^29^. The 1,536-well FRET assays to monitor HCV NS3-4A or human Furin activities were performed as follows: two µL enzyme solution (5 or 40 nM for NS3-4A and Furin, respectively) in assay buffer (20 mM Tris⋅HCl pH 8.0, 150 mM NaCl, with 0.01% Tween 20) were dispensed into a 1,536-well solid-bottom black plate (Greiner Bio One). 20 nL of compounds, positive control Aprotinin (50 µM), or neutral control DMSO were transferred via Echo 650 liquid handler (Beckman Coulter). Samples were incubated (RT, protected from light) for 15 min followed by addition of 2 µL of 0.625 µM substrate (Ac-DE-[Lys(5-TAMRA)]-EE-[Abu-psi(COO)Ala]-S-[Lys(ECLIPSE-7)]-amide or pERTKR-AMC for NS3-4A and Furin, respectively) in assay buffer. Plates were immediately read at time 0 and after 15 min incubation on a ViewLux high-throughput CCD imager (PerkinElmer) equipped with TAMRA (525 nm excitation, 598/25 nm emission) or AMC (355 nm excitation, 460 nm emission) fluorescence optics. The change in fluorescence intensity over the 15-min reaction period was normalized against DMSO and inhibitor controls as described below. Compounds were tested at 11 concentrations, 1:3 dilution points from 25 µM to 0.42 nM.

### Cell culture

HEK293T cells were obtained from ATCC (CRL-1573) and cultured in DMEM (4.5 g/L glucose) with 10% fetal bovine serum (FBS), 6 mM L-glutamine, 1 mM sodium pyruvate, 50 U/mL penicillin, and 50 µg/mL streptomycin. SNB-19 cells were grown in RPMI-1640 (cat# 11,875,093, Gibco) supplemented with 1% NEAA (cat# 11,140,050, Gibco) and 5% FBS. All cells were maintained in a humidified incubator at 37 °C and 5% CO_2_.

### Cell-based nsP2 assay

Construct cloning was produced by gene synthesis (GeneScript). The split Nluc reporter construct was cloned into the NheI/EcoRI sites of pcDNA3.1 ( +). The Nterm HiBiT (86b) and Cterm LgBiT (11S) sequences of the split Nluc reporter^34,49^ were separated by a flexible loop containing the nsp3/4 cleavage site (DELRLDRAGGYIFSSKGGGGSKL) or a flexible loop without a cleavage site (12X GlySer). nsP2pro expression construct (amino acids 220–798, accession #MT933050) was cloned into the NheI/EcoRI sites of pcDNA3.1 ( +) and contained a C-terminal Flag/6xHis tag. The gene synthesis of the split Nluc reporter and West Nile Virus (WNV) NS2B/NS3 protease expression constructs was performed by Synbio Technologies. The plit Nluc reporter construct included the N-terminal HiBiT (86b) and C-terminal LgBiT (11S) sequences, separated by a loop containing the WNV NS2B/NS3 cleavage site (LQYTKRGGVLGGGSKL). The WNV NS2B/NS3 protease expression construct (amino acids 1375–1693, accession #NC_009942) contained a C-terminal Flag/6xHis tag. Both constructs were cloned into the XbaI/AgeI sites of pcDNA3.4. For the 1,536-well assay, cells were transfected in T75 flasks using a reverse transfection procedure, where 9 mL of complexes containing 45 µL Lipofectamine 2000 (ThermoFisher Scientific), 20 µg nsP2 plasmid, and 0.2 µg Nluc reporter plasmid, were combined with 10 mL of HEK293T cell suspension (1 × 106 cells/mL, 10 million cells total). Complexes containing Transfection Carrier DNA (Promega) and Nluc reporter plasmid (also at 1:100 ratio) were used as a high-signal control. After 24 h, cells were harvested by trypsinization and resuspended at a density of 1 × 10^6^ cells/ml. Cells were dispensed (4 µL/well) into 1,536-well white plates (Aurora, cyclic olefin polymer, cat# EWB041000A) using a Multidrop Combi. After 4 h, compounds (20 nL) were subsequently dispensed using an Echo 650 Series liquid handler and incubated for overnight at 37 °C. Nano-Glo reagent was added (4 µL /well) per manufacturer’s recommendations. The plates were centrifuged and analyzed for luminescence intensity using a ViewLux reader. For cell viability measures shown in Supplementary Fig. 10A-B, 3 µL /well of CellTiter-Glo (Promega) was added instead of Nano-Glo reagent and analyzed for luminescence intensity as described above.

### Cell viability assay of SNB19 cells

Cells were harvested in growth media and dispensed (5 µL /1,000 cells/well) onto 1536-well white plates (Aurora #EWB041000A) using a Multidrop Combi. Control and test compounds were added (25 nL) using an Echo 650 liquid handler. Plates were incubated for 48 h at 37 °C and 5% CO_2_ and then equilibrated to room temperature prior to addition of 4 µL /well of CellTiter-Glo (Promega) using a Multidrop Combi. Plates were subsequently incubated for 15 min at room temperature and centrifuged for 1 min at 1000 rpm. Luminescence was read on a ViewLux imager equipped with clear filters.

### Virus stock preparation

Chikungunya virus (CHIKV) strain 181/25, biosafety level 2 (BSL-2) was obtained from BEI resources (# NR-56523). All viruses were handled in level II biosafety cabinets in a biosafety level 2 laboratory according to approved NIH Biosafety Protocols. Briefly, to prepare the virus stocks for CHIKV, Vero E6 cells (ATCC, # CRL-1586) were cultured in DMEM (Gibco cat# 11,965,092) containing 5% FBS (Gibco, #A5670701) at 37 °C in 5% CO_2_ until the cell monolayer reached approximately 80% flask confluency. Cells were subsequently infected with a virus inoculum of 0.1 MOI at low volume (5 mL in T-75 flask), cells were kept rocking at 37 °C for 1 h, then 15 mL of fresh 5% FBS DMEM was added, and flasks were placed back in the incubator at 37 °C for 1–3 days. Plates were observed for cytopathic effect (CPE) during the culture and once signs of CPE reaching approximately 50% of the cell monolayer were observed, supernatant was collected and filtered through a 0.22 µm filter unit, aliquoted and stored at -80 °C. Viral titers were quantified using standard 50% tissue culture infectious dose (TCID_50_) assays in Vero E6 cells. Briefly, Vero E6 cells were seeded on 384-well plates at 5000 cells/well and cultured in DMEM with 5% FBS overnight in a 37 °C, 5% CO_2_ incubator. For each sample, serial dilutions were performed and 30 µL of diluted samples were added to each well in triplicates to follow a 1:10 dilution scheme. Plates were incubated at 37 °C, 5% CO_2_ for 48 h. After incubation, cells were fixed for 15 min in 4% paraformaldehyde. Fixed cells were then permeabilized with 0.3% PBT (1X PBS, 0.3% Triton-X) then blocked with PBTG blocking buffer (1X PBS with 0.1% Triton X-100, 1% bovine serum albumin (BSA) and 5% normal goat serum) for 1 h at room temperature and then incubated with anti-Chikungunya virus antibody clone 6A11 (Millipore Sigma cat#MABF2051) in 1:1000 dilution using blocking buffer. Plates in primary antibody solution were incubated overnight at 4 °C followed by 3 washes of 1X PBS. Cells were next incubated for 1 h at room temperature with Goat anti-Mouse IgG (H + L) Cross-Adsorbed Secondary Antibody, Alexa Fluor™ 488 (ThermoFisher, # A-11001) at 1:1000 dilution. After 3 washes with 1X PBS, 50 μL of 1X PBS was dispensed and plates were imaged on an automated HT Opera Phenix (Revvity) to detect infected cells in cell monolayer.

### Viral assays

The live virus cell-based assay performed is detailed in Medina et al. (manuscript in preparation). In short, SNB-19 cells were seeded in 1536-well plates suitable for high-content imaging at 750 cells/well in RPMI media supplemented with fetal bovine serum (5% FBS) and non-essential amino acids (1X NEAA) and incubated overnight. Cells were exposed to test compounds by acoustic dispense on the pre-seeded plates with an Echo 655 and incubated for an hour prior to virus inoculation. The CHIKV 181/25 vaccine strain was used at an MOI of 1 and cells were incubated at 37 °C, 5% CO_2_ for 44 h. After incubation, cells were fixed with 4% PFA for 20 min, permeabilized and stained for immunofluorescence assay and imaging using the Opera Phenix High-Content Screening System (Revvity). Nuclei count and percentage of infected cells was calculated after image analysis using Columbus Scope software by PerkinElmer.

### qHTS data analysis

Data from each assay were normalized plate-wise to corresponding DMSO and positive controls as described previously^50^. The same controls were also used for the calculation of the Z’ factor. Supplementary Fig. 14 contains Z’ and S/B values for all primary and follow-up screens. Percent activity was derived and fitted to the Hill equation using inhouse software (http://tripod.nih.gov/curvefit/). All concentration–response curves (CRCs) were classified as described previously^22,50^. For primary and follow-up screens utilizing nsP2pro, compounds exhibiting high-quality CRCs (class -1 and -2), efficacy > 50%, and IC_50_ < 10 µM were considered active. The same criteria were applied to characterize activity in other protease assays except no IC_50_ cut off was applied. For cell-based reporter assays, compounds displaying a positive curve class 1 or 2 and efficacy > 50% were considered active. For viral assays, compounds displaying curve class -1 or -2, efficacy > -50% and inactive in cytotoxic assays or with an IC_50_ ten-fold lower than in cytotoxic assays were considered active. Structure clustering (UPGMA method) and heat maps were generated in TIBCO Spotfire (TIBCO Software Inc., Palo Alto, CA). All other graphs were generated in GraphPad Prism (GraphPad Software, Boston, MA).

### Molecular docking

The protein structure of CHIKV nsP2pro, SARS-CoV-2 PLpro, and Papain were obtained from the Protein Data Bank (PDB 3TRK, 6WUU, and 6TCX, respectively). Prior to docking, the 3D structure was processed using the Structure Preparation Module in the MOE program (www.chemcomp.com). Docking studies of the identified small molecule inhibitors to the active site of nsP2pro were performed using the MOE Dock. The ligand induced fit docking protocol was applied, and binding affinity was calculated using the GBVI/WSA score. The top-ranked docking poses were inspected and further minimized and re-scored. The best predicted binding model with the lowest binding affinity was selected for structural binding analysis.

## Supplementary Information

Supplementary Information.

## Data Availability

All qHTS data generated is made available to the scientific community through PubChem (AID 1,964,100-1,964,107): https://pubchem.ncbi.nlm.nih.gov/bioassay/1964100; https://pubchem.ncbi.nlm.nih.gov/bioassay/1964101; https://pubchem.ncbi.nlm.nih.gov/bioassay/1964102; https://pubchem.ncbi.nlm.nih.gov/bioassay/1964103; https://pubchem.ncbi.nlm.nih.gov/bioassay/1964104; https://pubchem.ncbi.nlm.nih.gov/bioassay/1964105; https://pubchem.ncbi.nlm.nih.gov/bioassay/1964106; https://pubchem.ncbi.nlm.nih.gov/bioassay/1964107.
