# Supplementary material for: A quantitative high-throughput screening pipeline to identify small molecule inhibitors of Chikungunya nsP2 protease
Source: Sci Rep. 2025 Sep 29;15:33479. doi: 10.1038/s41598-025-14697-3 (PMC12480751; doi:10.1038/s41598-025-14697-3)
Supplement: Supplementary file 1 — Supplementary Information. [file 41598_2025_14697_MOESM1_ESM.pdf]

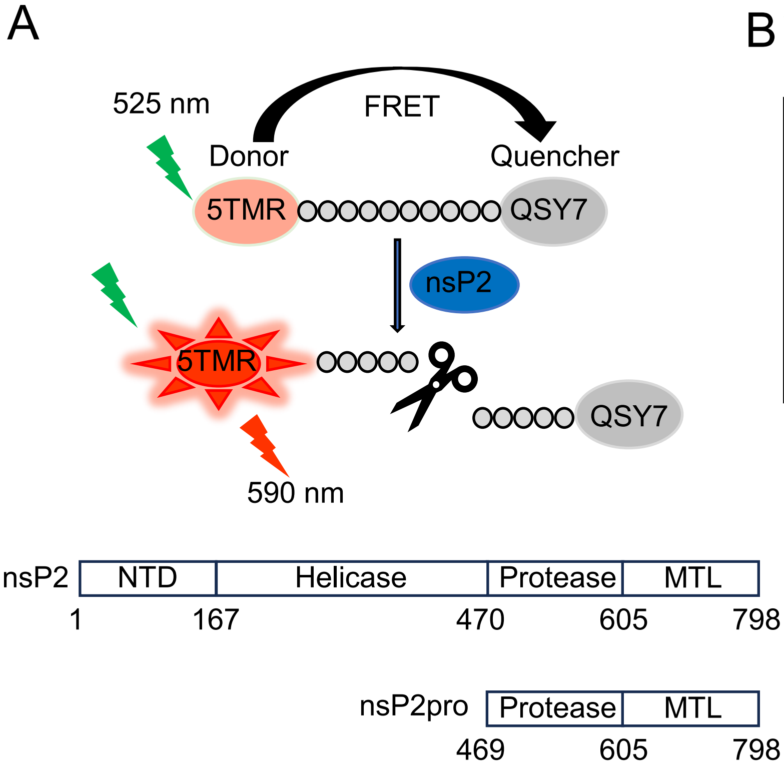

Peptide 1 (nsp3/4): 5TMR-RAGG/YIFS-K-QSY7
Peptide 2 (nsp3/4): 5TMR-DELRLDRAGG/YIFSS-K-QSY7
Peptide 3 (nsp2/3): 5TMR-DELRLDRAGC/APSYR-K-QSY7

B

		Peptide 1	Peptide 2	Peptide 3
nsP2pro	K_m [µM]	ND	6.3 ± 0.3	81.9 ± 9.2
	kcat [x 10^{-4} sec^{-1}]	ND	214.2 ± 13.1	85.5 ± 4.1
	kcat/Km [x 10^{-4} $µM^{-1}$ sec^{-1}]	ND	34.1 ± 1.9	1.1 ± 0.1
FL nsP2	K_m [µM]	ND	1.1 ± 0.1	37.5 ± 2.0
	kcat [sec^{-1}]	ND	167.2 ± 10.4	32.1 ± 2.1
	kcat/Km [$µM^{-1}$ sec^{-1}]	ND	157.8 ± 3.8	0.86 ± 0.1

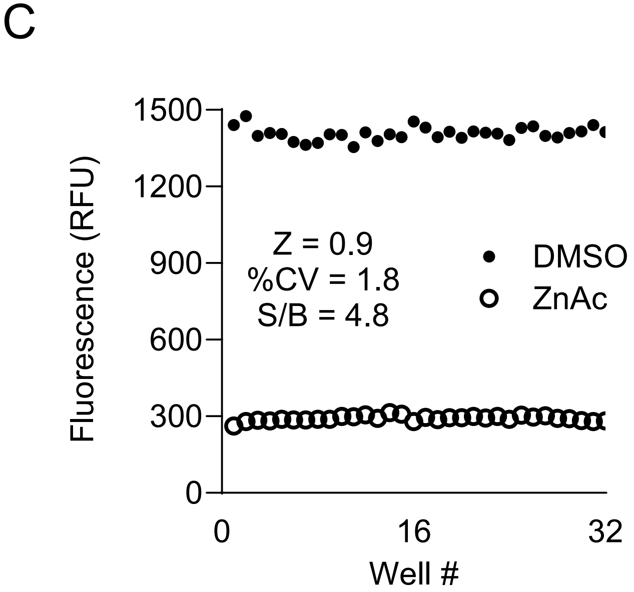

Li et al. Figure 1

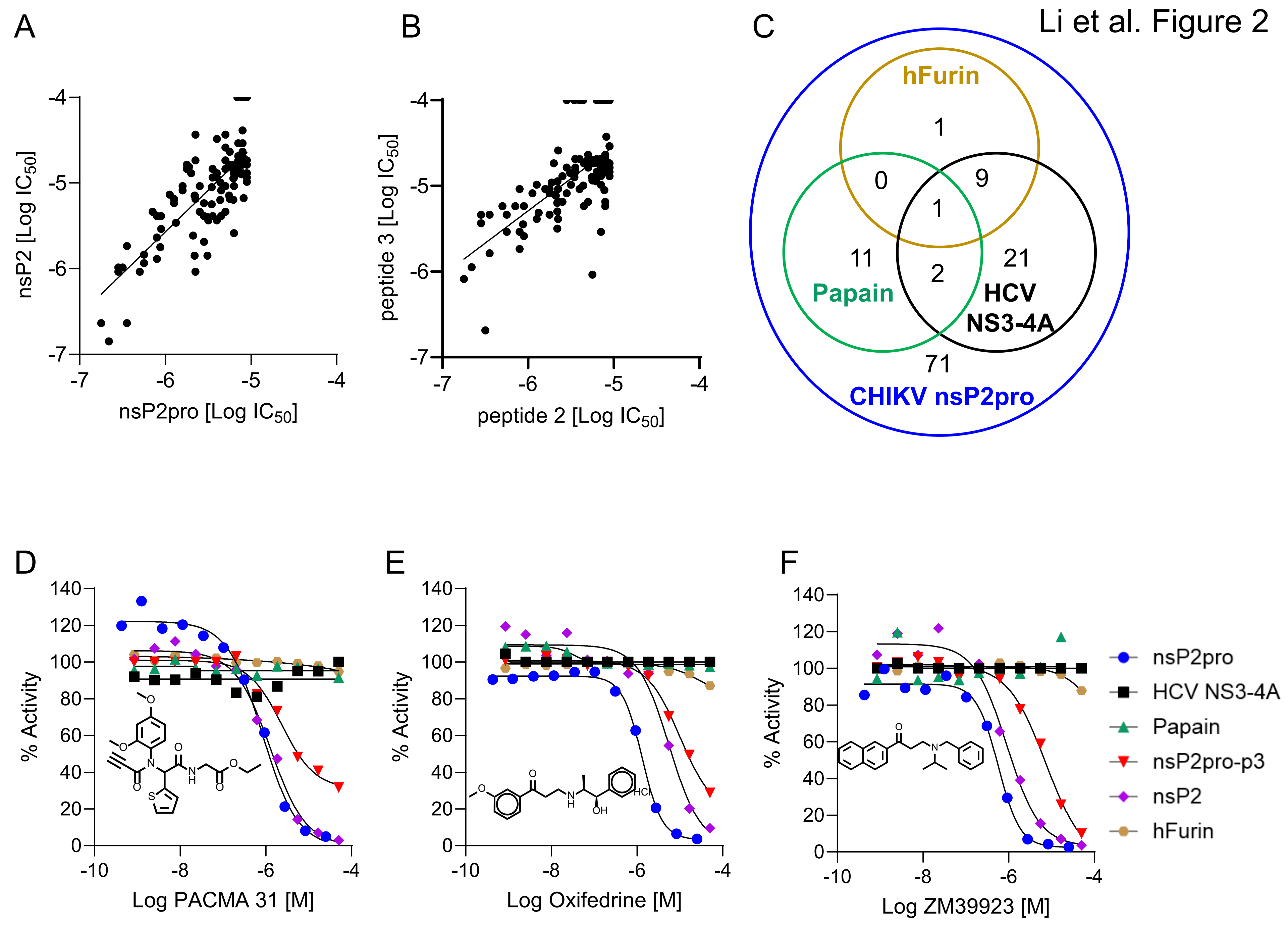

Li et al. Figure 2
A
nsP2 [Log IC50]
nsP2pro [Log IC50]
B
peptide 3 [Log IC50]
peptide 2 [Log IC50]
C
hFurin
1
0
9
1
11
2
21
Papain
HCV NS3-4A
71
CHIKV nsP2pro
D
% Activity
Log PACMA 31 [M]
E
% Activity
Log Oxifedrine [M]
F
% Activity
Log ZM39923 [M]
nsP2pro
HCV NS3-4A
Papain
nsP2pro-p3
nsP2
hFurin

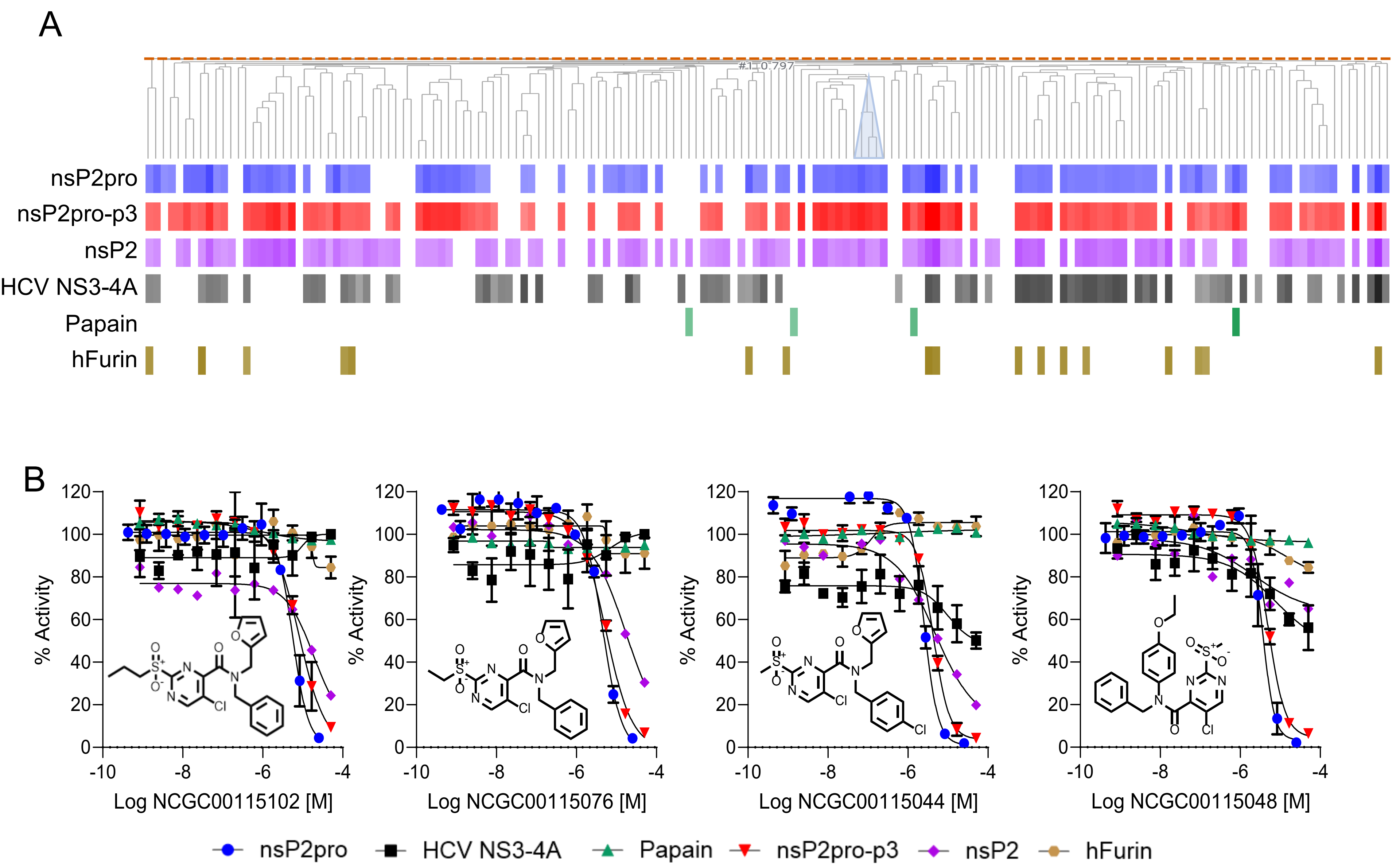

Li et al. Figure 3

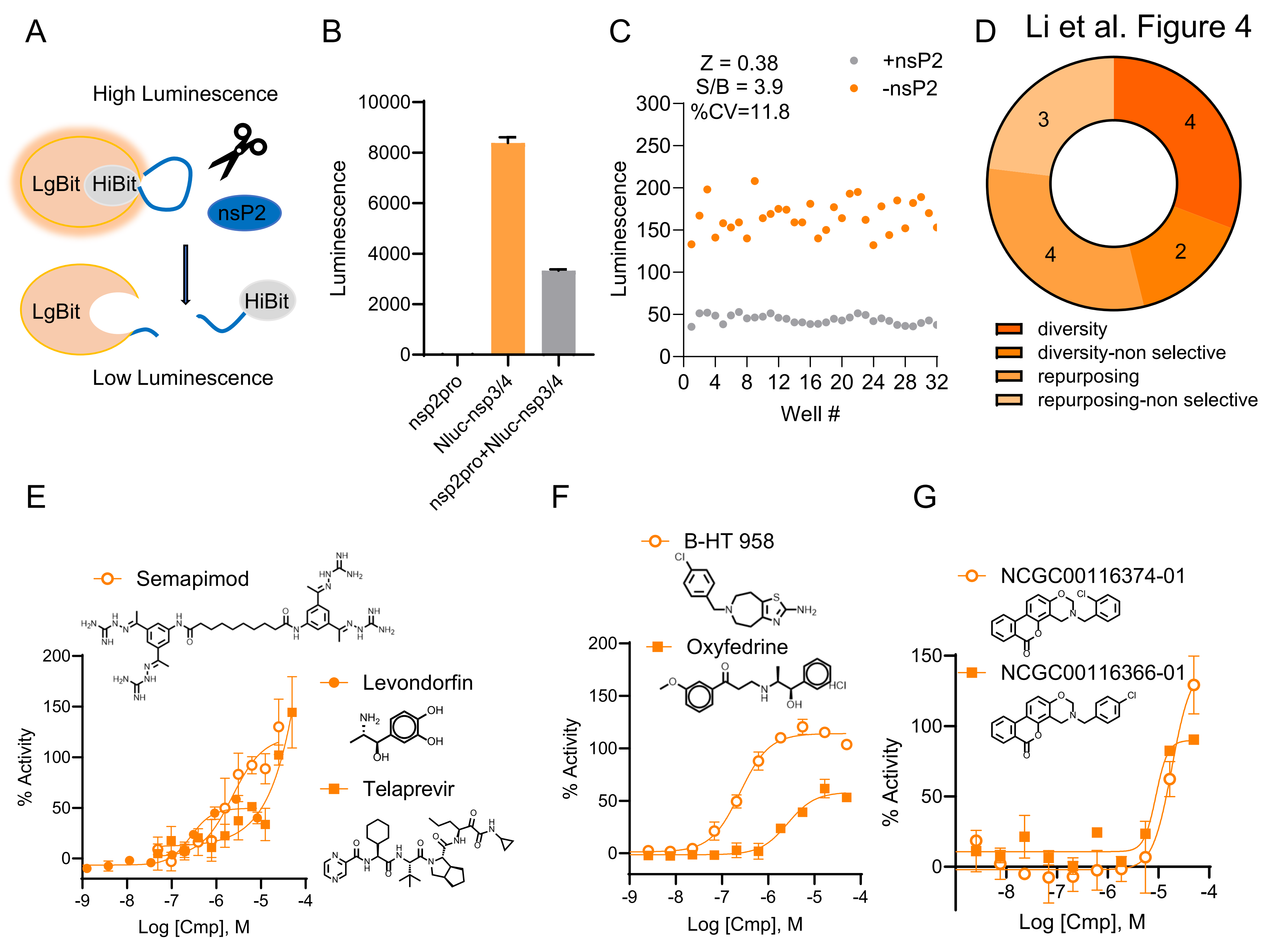
Li et al. Figure 4
A
High Luminescence
LgBit
HiBit
nsP2
Low Luminescence
B
Luminescence
nsp2pro
Nluc-nsp3/4
nsp2pro+Nluc-nsp3/4
C
Z = 0.38
S/B = 3.9
%CV=11.8
+nsP2
-nsP2
Luminescence
Well #
D
diversity
diversity-non selective
repurposing
repurposing-non selective
E
Semapimod
Levondorfin
Telaprevir
% Activity
Log [Cmp], M
F
B-HT 958
Oxyfedrine
% Activity
Log [Cmp], M
G
NCGC00116374-01
NCGC00116366-01
% Activity
Log [Cmp], M

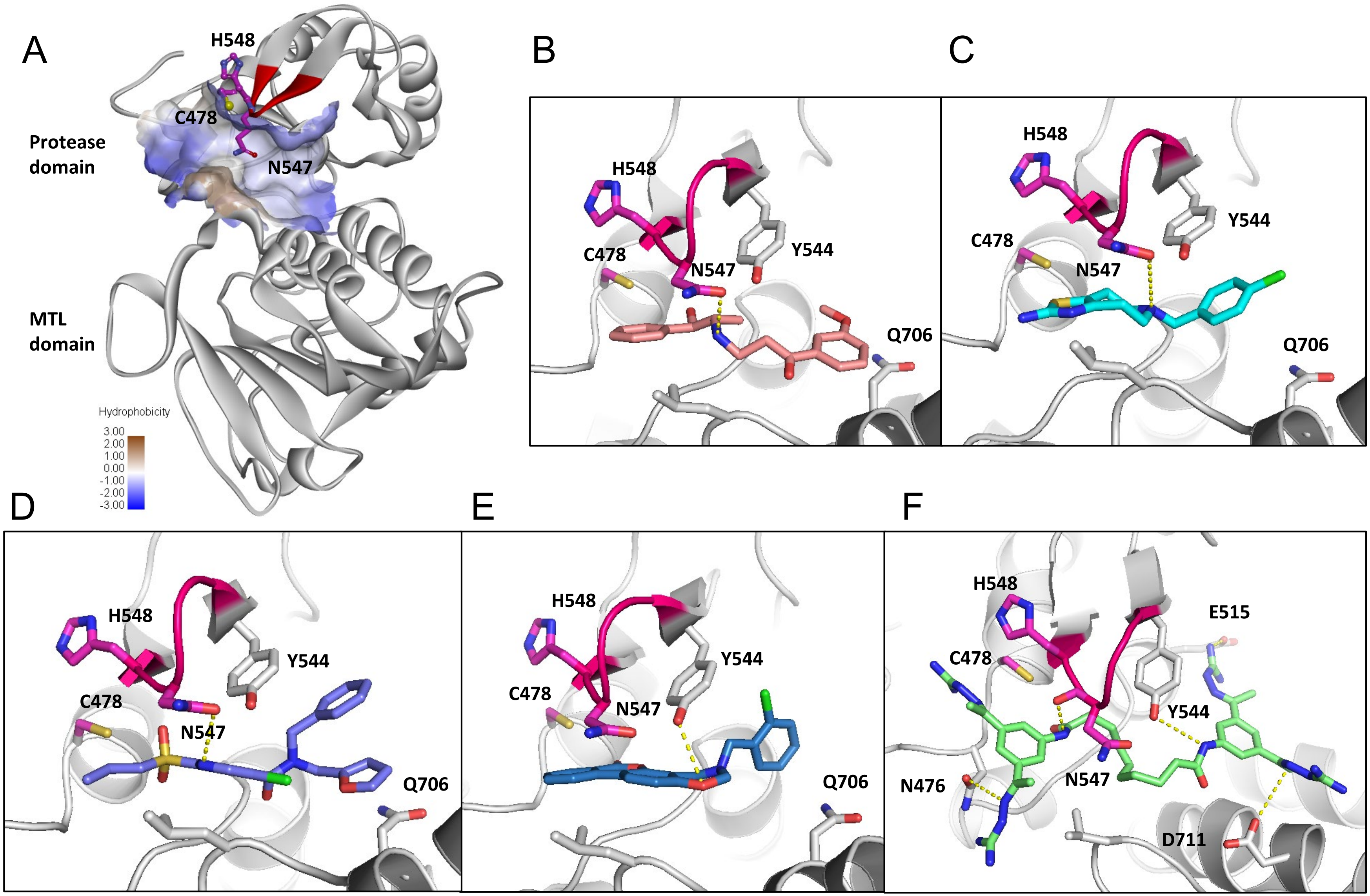

Li et al. Figure 5

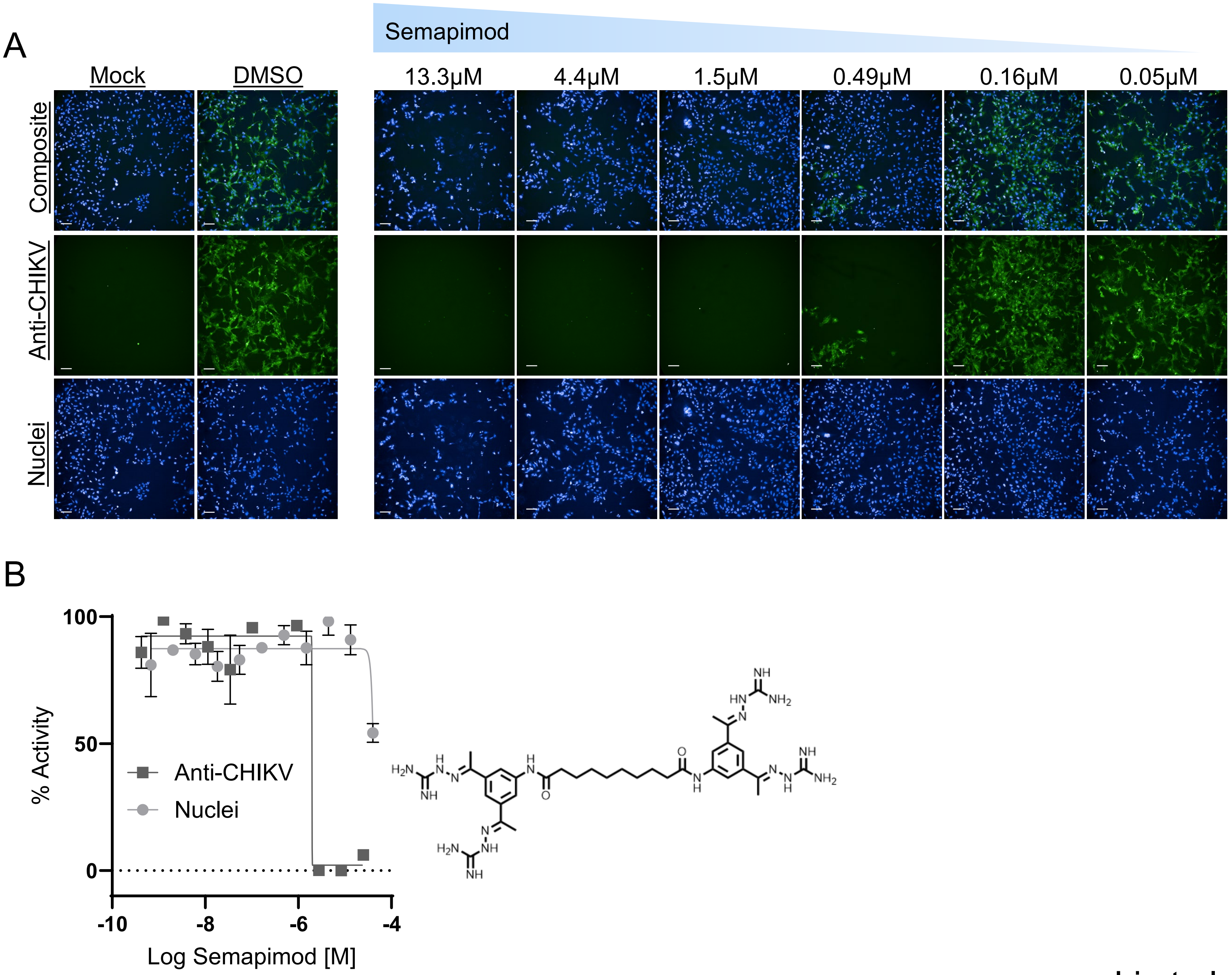

Li et al. Figure 6

Supplementary Figures

A quantitative high-throughput screening pipeline to identify inhibitors of Chikungunya nsP2 protease

Shuaizhang Li[1], Xin Hu[1], Yong-Mo Ahn[1], Angelica Medina[1], Lin Ye[1], Audrey Heffner[1], Simon Messing[2], John-Paul Denson[2], Dominic Esposito[2], Emily M. Lee[1], and Natalia J. Martinez[1]*

[1]National Center for Advancing Translational Sciences, National Institutes of Health, Rockville, MD, USA
[2]Protein Expression Laboratory, Cancer Research Technology Program, Frederick National Laboratory for Cancer Research, Frederick, MD, USA

*Corresponding author (natalia.martinez@nih.gov)

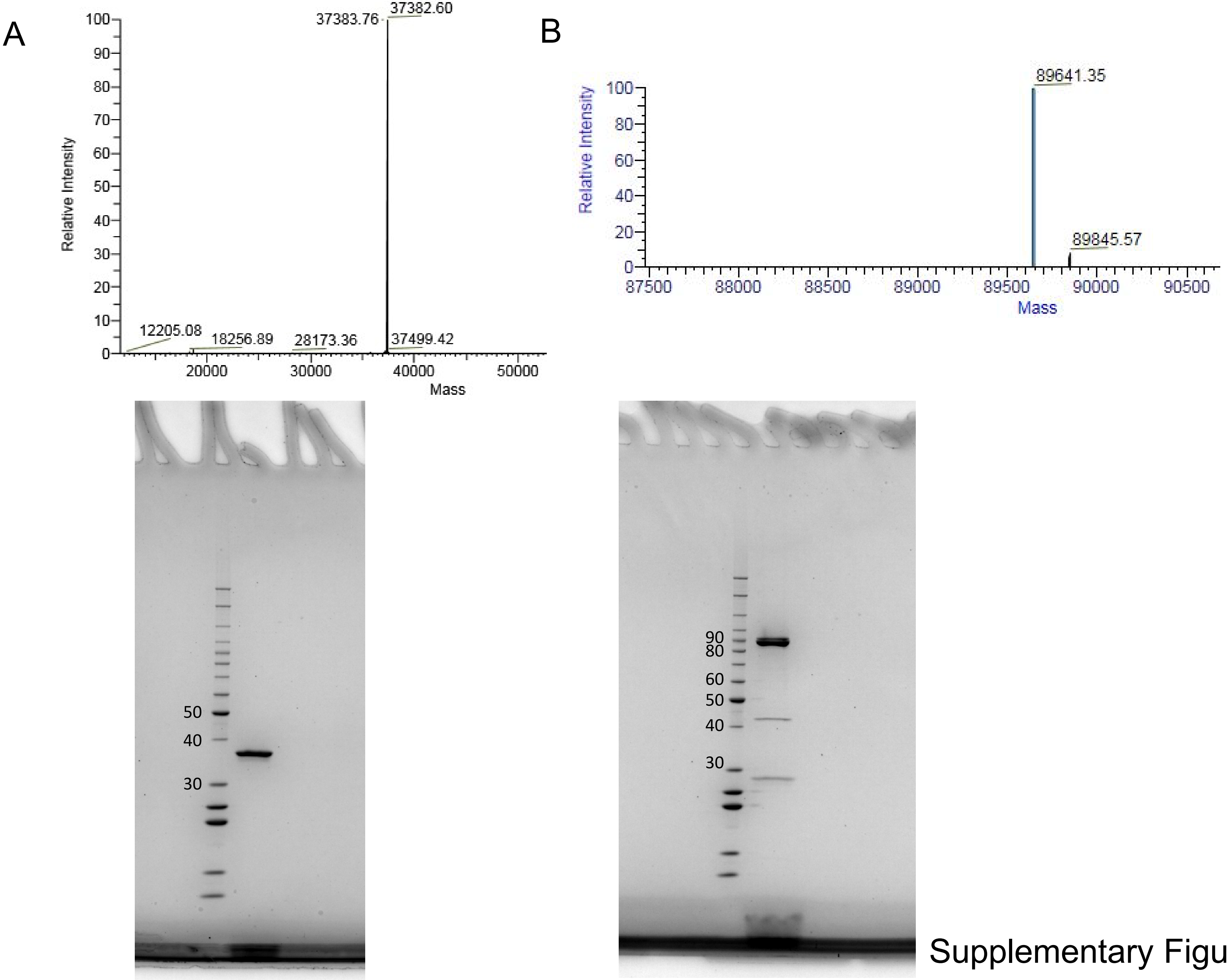

Supplementary Figure 1

Supplementary Figure 1. Expression and purification of recombinant protease domain and full-length nsP2. Shown are SDS-PAGE gels and ESI-MS analysis of the final purified CHIKV nsP2pro (aa 469-798) (A), and full length CHIKV nsP2 (aa 1-798) (B). SDS-PAGE markers are denoted in kilodaltons. Expected molecular masses of the two proteins are 37,384 Da (A) and 89,642 Da (B). Estimated purity based on densitometry is >95% for nsP2pro and 82% for full length nsP2.

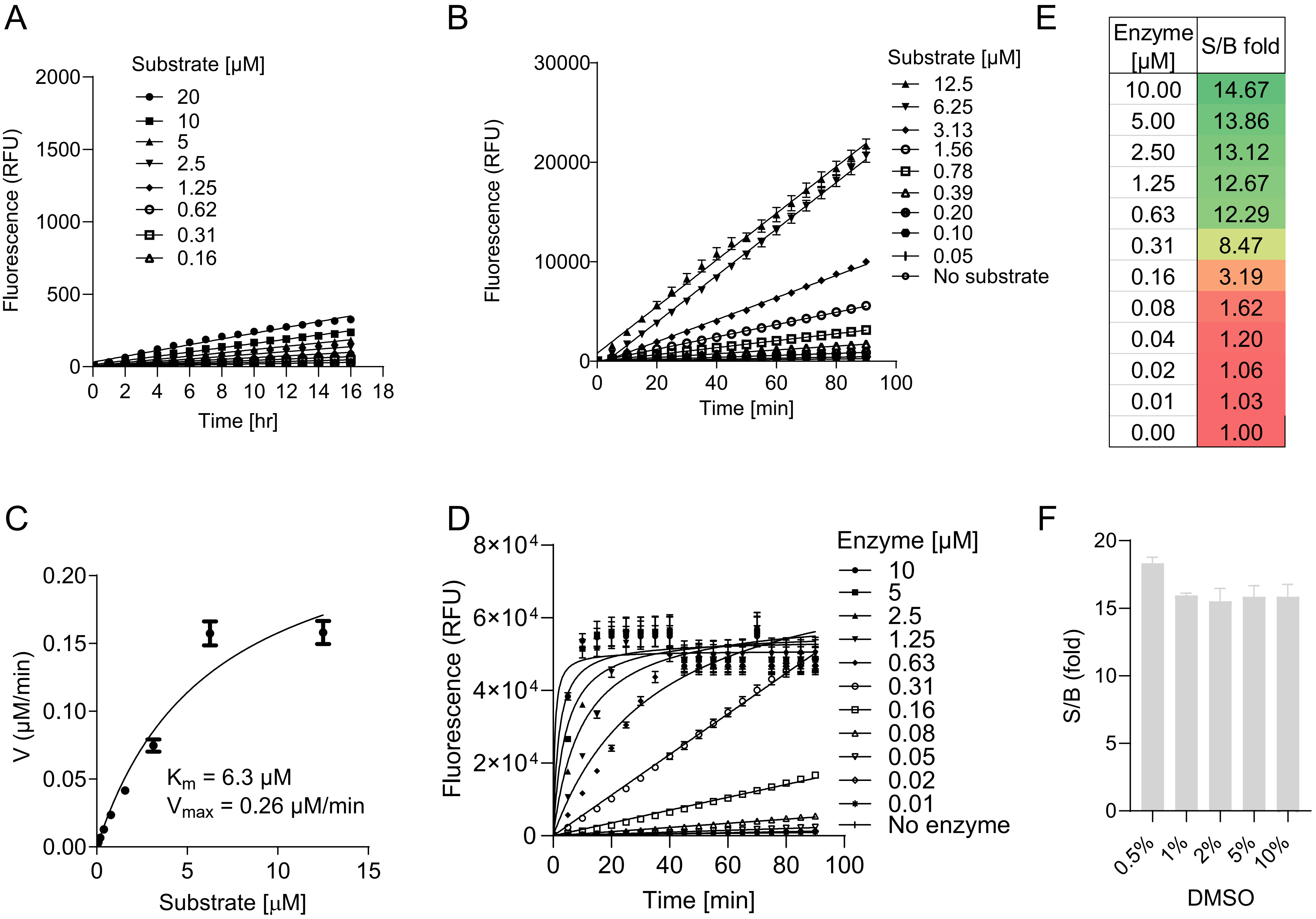

Supplementary Figure 2

Supplementary Figure 2. nsP2pro assay development using peptides 1 and 2 as substrates. (A) Reaction progress curves determined using indicated concentrations of peptide 1 (10 aa long) and 1 μM nsP2pro. (B) Reaction progress curves determined using indicated concentrations of peptide 2 (15 aa long) and 150 nM nsP2pro. (C) Enzymatic kinetic parameters determined by fitting initial velocities of reactions containing 150 nM nsP2pro and varying concentrations of peptide 2 to the Michaelis-Menten equation. K_m and V_{max} are indicated. (D) Progress curve analysis for different concentration of nsP2pro at a fixed 5 μM concentration of peptide 2. (E) Signal-to-background (S/B) ratios of reactions containing 5 μM peptide 2 and indicated concentrations of nsP2pro at 1 h incubation. (F) DMSO sensitivity: Signal-to-background (S/B) ratios of 1 hr reactions determined using 5 μM substrate, 150 nM nsP2pro enzyme, and indicated DMSO concentrations. In all panels, raw fluorescence units (RFU) were corrected by subtracting background fluorescence at time 0. Data represents average +/- SDof n=3, except for panel A in which n=2.

A

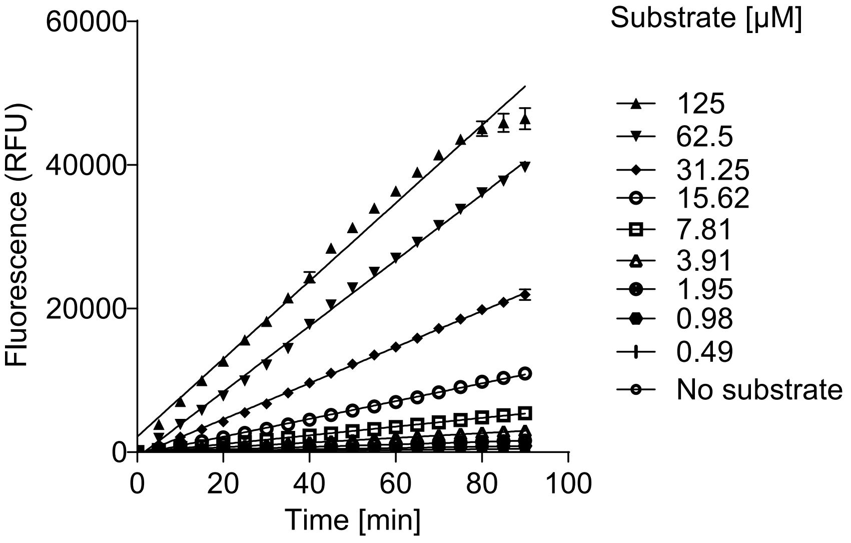

B

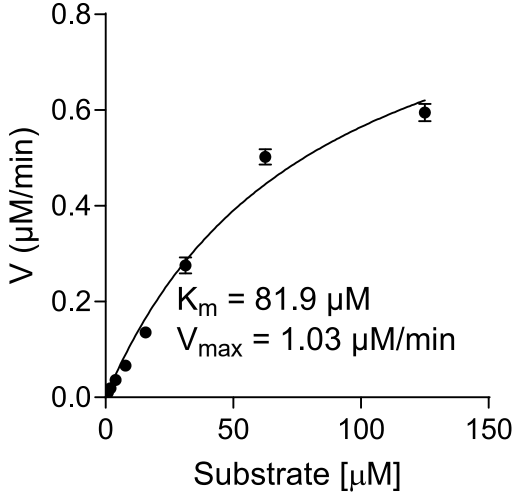

C

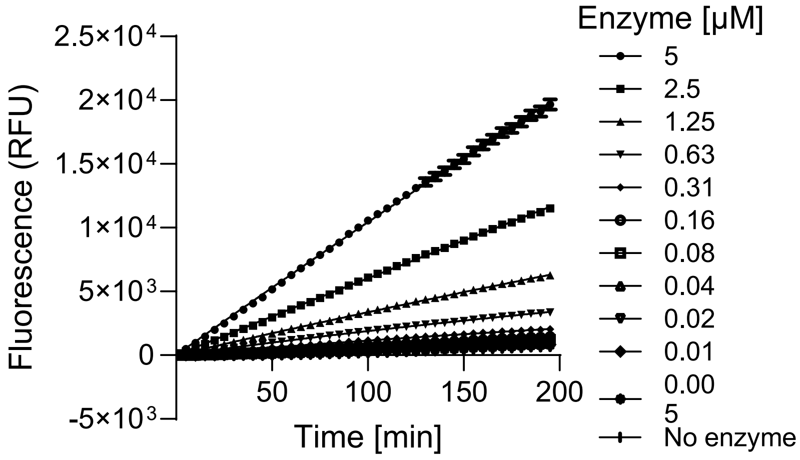

D

Enzyme [µM]	S/B fold
5.00	4.06
2.50	2.72
1.25	2.03
0.63	1.59
0.31	1.36
0.16	1.24
0.08	1.14
0.04	1.10
0.02	1.05
0.01	1.03
0.005	1.00
0.00	1.00

Supplementary Figure 3

Supplementary Figure 3. nsP2pro cleavage of nsp2/3 substrate (peptide 3). (A) Reaction progress curves determined using indicated concentrations of peptide 3 (15 aa long) and 2 µM nsP2pro. (B) Enzymatic kinetic parameters determined by fitting initial velocities of reactions containing 2 µM nsP2pro and varying concentrations of peptide 3 to the Michaelis-Menten equation. K_m and V_{max} are indicated. (C) Progress curve analysis for different concentration of nsP2pro at a fixed 5 µM concentration of peptide 3. (D) Signal-to-background (S/B) ratios at 5 µM peptide 3 and indicated concentrations of nsP2pro at 1 h incubation. In all panels, raw fluorescence units (RFU) were corrected by subtracting background fluorescence at time 0. Data represents average of n=3 +/- SD.

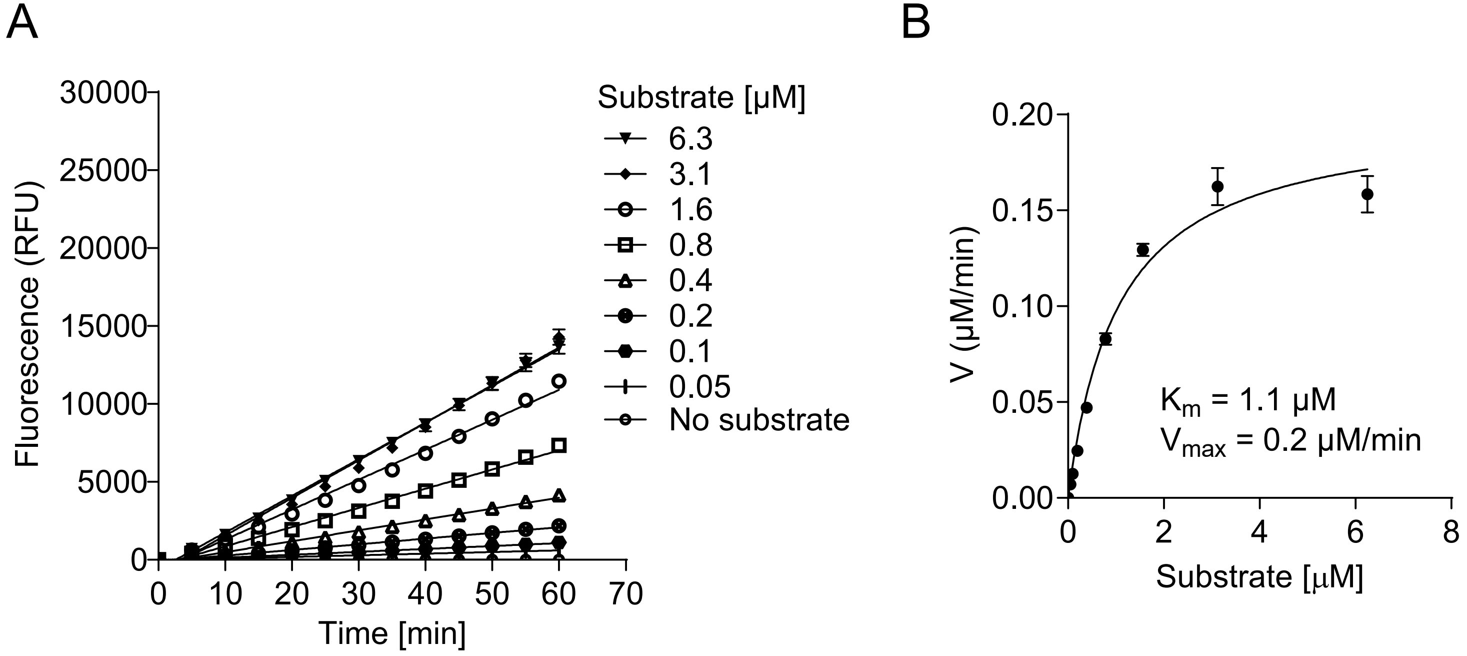

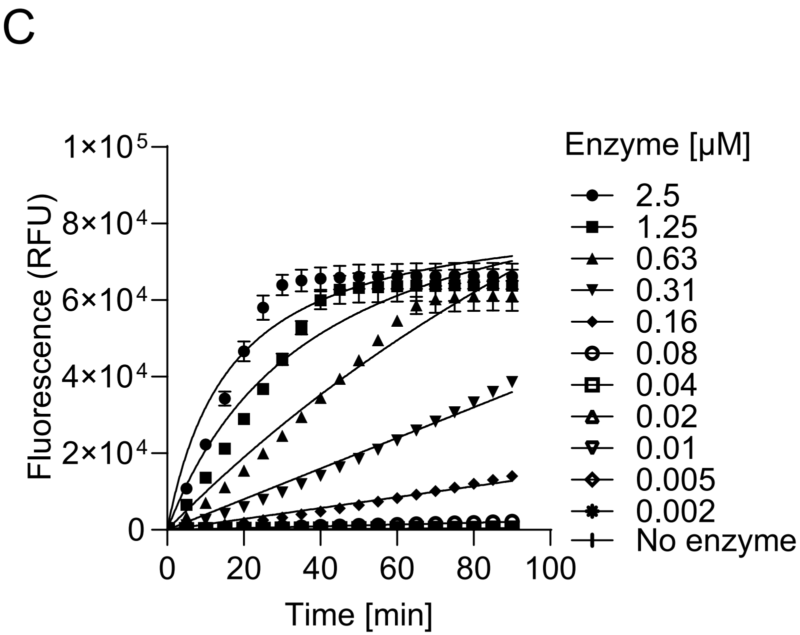

D

Enzyme [µM]	S/B fold
2.50	123.7
1.25	116.6
0.63	98.9
0.31	42.7
0.16	15.7
0.08	3.4
0.04	1.5
0.02	1.2
0.01	1.1
0.005	1.0
0.002	1.0
0.00	1.0

Supplementary Figure 4

Supplementary Figure 4. Full-length nsP2 cleavage of nsp3/4 (peptide 2) substrate. (A) Reaction progress curves determined using indicate concentrations of peptide 2 (15 aa long) and 150 nM nsP2. (B) Enzymatic kinetic parameters determined by fitting initial velocities of reactions containing 150 nM full-length nsP2 and varying concentrations of peptide 2 to the Michaelis-Menten equation. K_m and V_{max} are indicated. (C) Progress curve analysis for different concentration of nsP2 at a fixed 5 µM concentration of peptide 2. (D) Signal-to-background (S/B) ratios at 5 µM peptide 2 and indicated concentrations of nsP2 at 1 h incubation. In all panels, raw fluorescence units (RFU) were corrected by subtracting background fluorescence at time 0. Data represents average +/- SD of n=3.

A

B

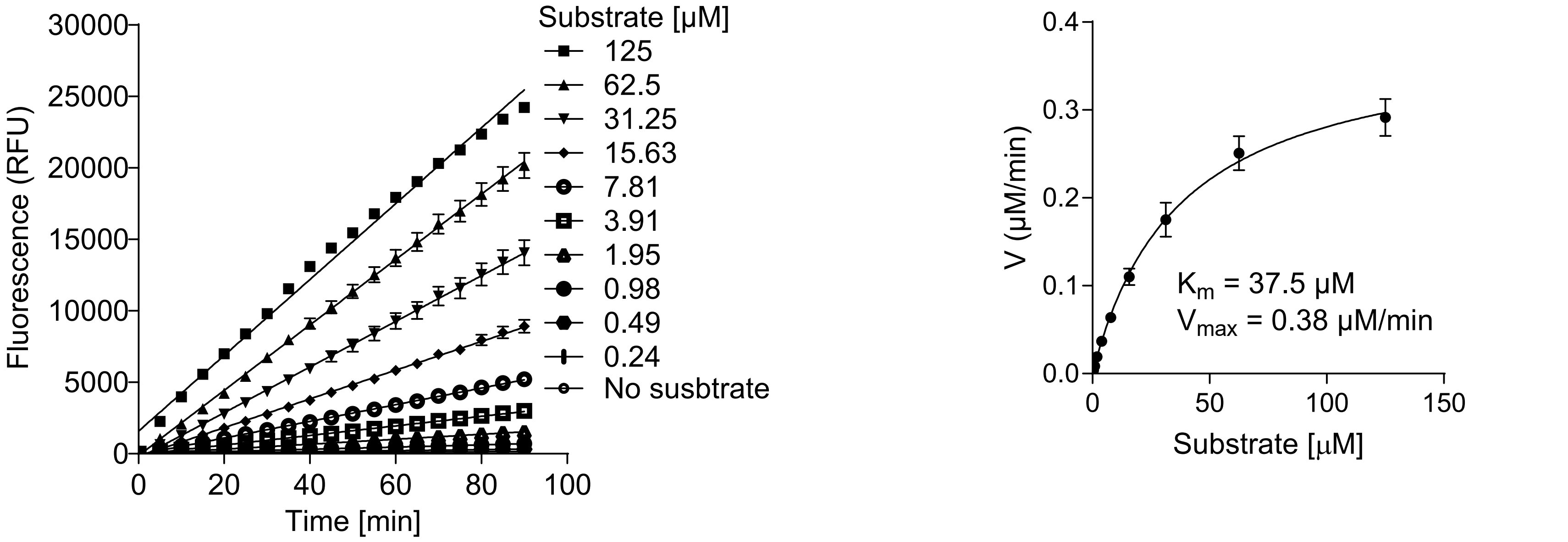

Supplementary Figure 5. Full-length nsP2 cleavage of nsp2/3 (peptide 3) substrate. (A) Reaction progress curves determined using indicated concentrations of peptide 3 (15 aa long) and 2 µM nsP2. (B) Enzymatic kinetic parameters determined by fitting initial velocities of reactions containing 2 µM full-length nsP2 and varying concentrations of peptide 3 to the Michaelis-Menten equation. K_m and V_{max} are indicated. In all panels, raw fluorescence units (RFU) were corrected by subtracting background fluorescence at time 0. Data represents average +/- SD of n=3.

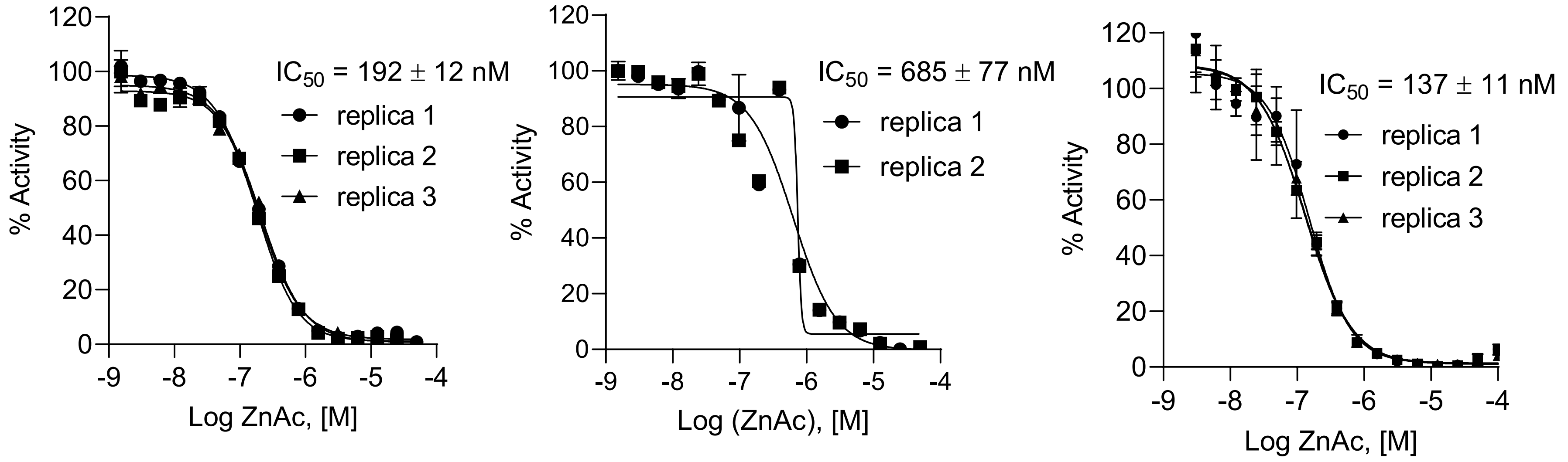

Supplementary Figure 6. Assay validation using ZnAc as inhibitor. Concentration-response curves of ZnAc inhibitor against nsP2pro and peptide 2 (left), nsP2pro and peptide 3 (middle), or full-length nsP2 and peptide 2 (right). For each replicate, data represents average +/- SD of n=3.

Supplementary Figure 6

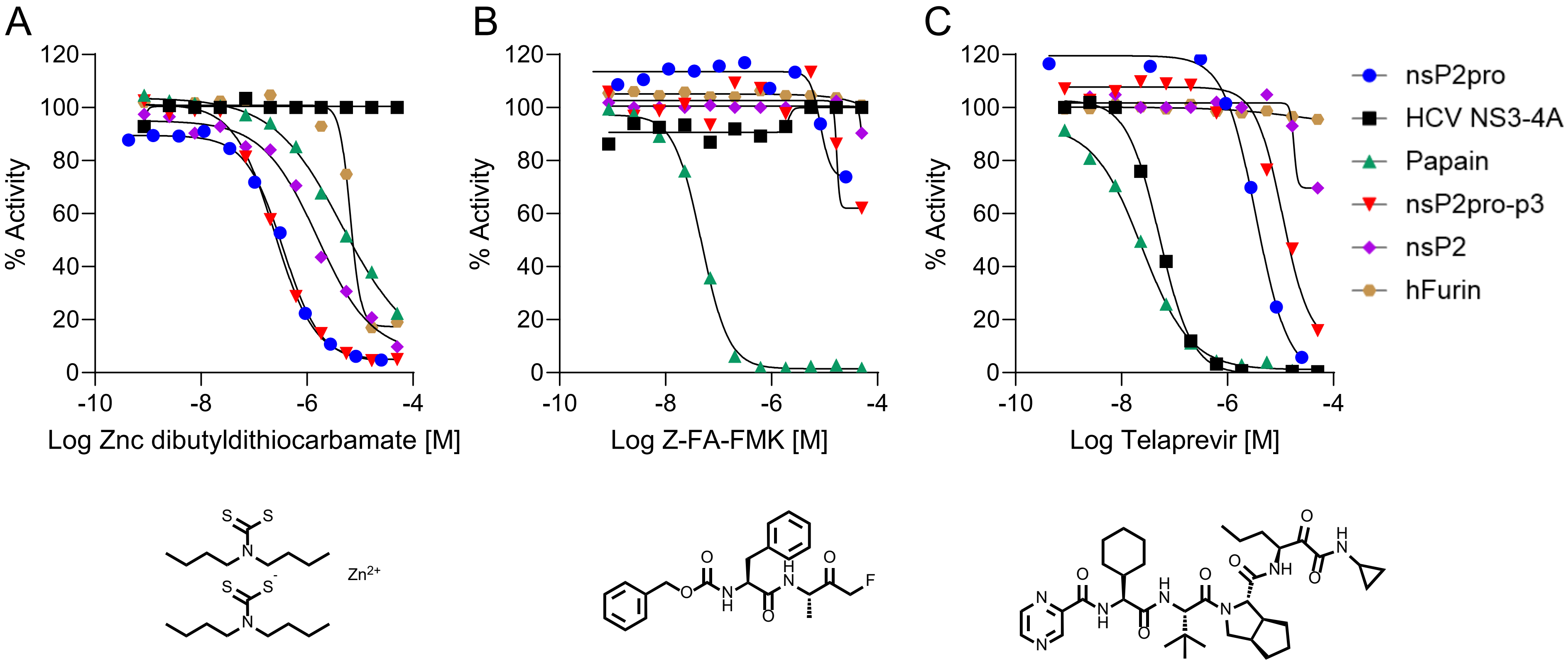

Supplementary Figure 7. Examples of non- selective or inactive hits. (A-C) Dose-response curves of compound activity in follow-up and counterscreen assays for (A) the non-selective Zinc dibutyldithiocarbamate, (B) the papain-selective Z-FA-FMK, and (C) the potent inhibitor of Papain and HCV NS3-4A but weak inhibitor of nsP2 Telaprevir. nsP2pro (blue), nsP2pro-peptide 3 (red), nsP2 full-length (purple), Papain (green), hFurin (brown), and HCV NS3-4A (black).

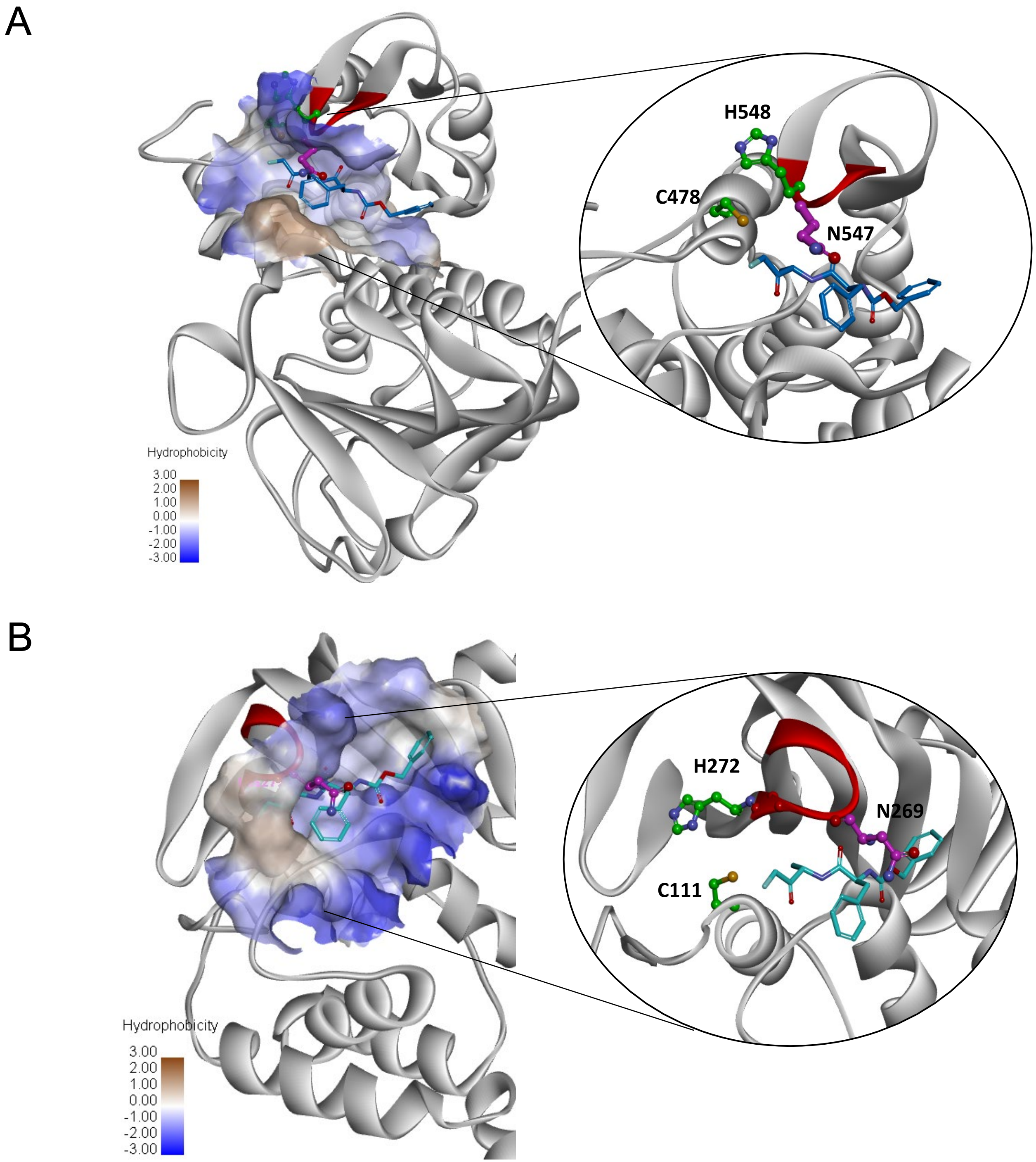

Supplementary Figure 8

C

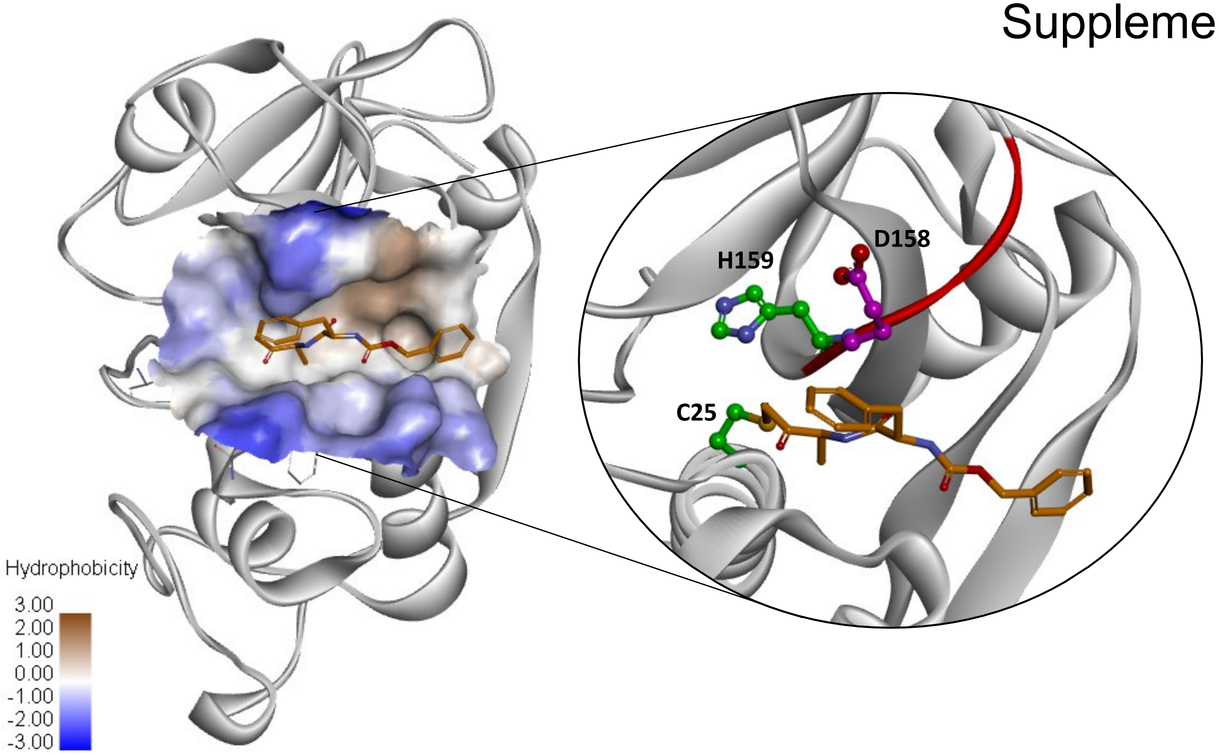

Supplementary Figure 8. Predicted binding models of Z-FA-FMK bound to the substrate binding site of (A) nsP2pro, (B) Plpro, and (C) Papain. Protein is shown in ribbon form with the active site in hydrophobic surface representation. Small-molecule inhibitors are shown in sticks. Circles zoomed into the catalytic pocket to highlight compound interaction. Z-FA-FMK likely does not fit into the active site pocket of nsP2pro (A, blue sticks) and PLpro (B, cyan sticks) to form covalent binding with the catalytic Cys. Residues Asn547 (nsP2pro) and Asn269 (PLpro) from the flexible DL loop (highlighted in red) are found to point to the active site and block the inhibitor binding. The active site of Papain (C) is more open, with an Asp158 at this position, which makes it amenable for Z-FA-FMK binding into the pocket and form covalent binding with Cys25.

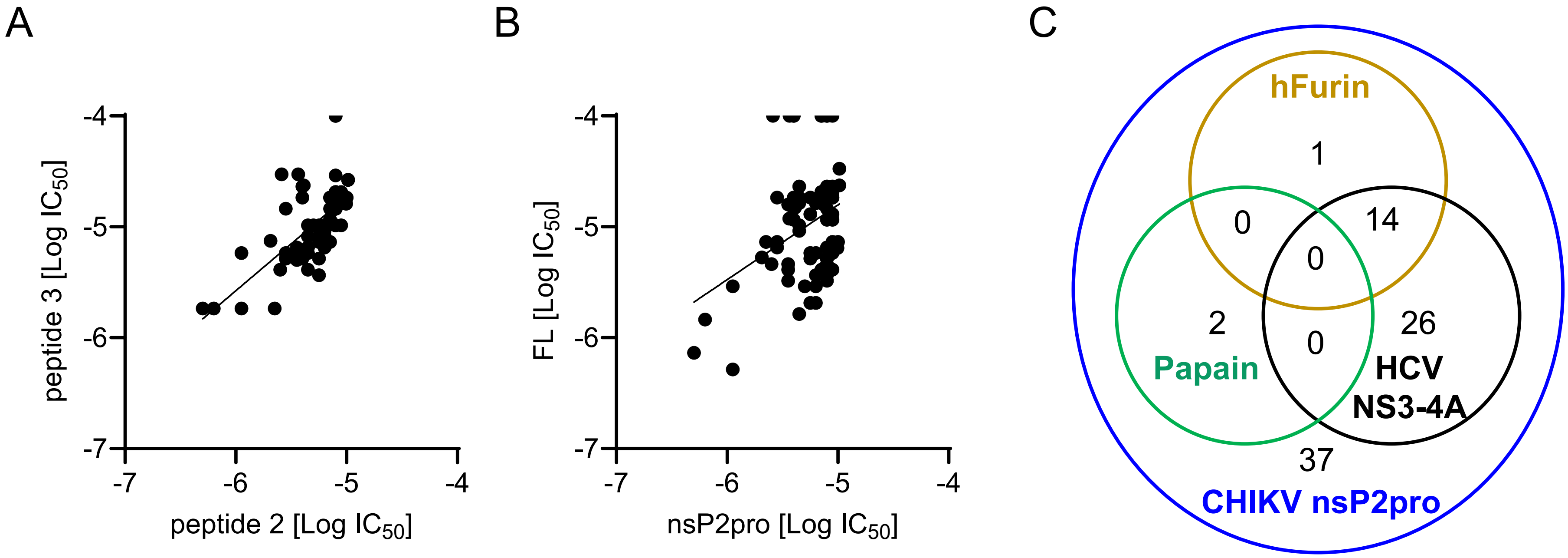

Supplementary Figure 9. Screening of diversity libraries. (A) Correlation plot of compound inhibitory activity against nsP2pro (Log IC_{50}) in peptide 2 vs. peptide 3 enzymatic reactions (R^2=0.46). (B) Correlation plot of compound inhibitory activity (Log IC_{50}) in truncated nsP2 protease domain vs. full-length nsP2 enzymatic reactions (R^2=0.12) using peptide 2 as substrate.

Supplementary Figure 9

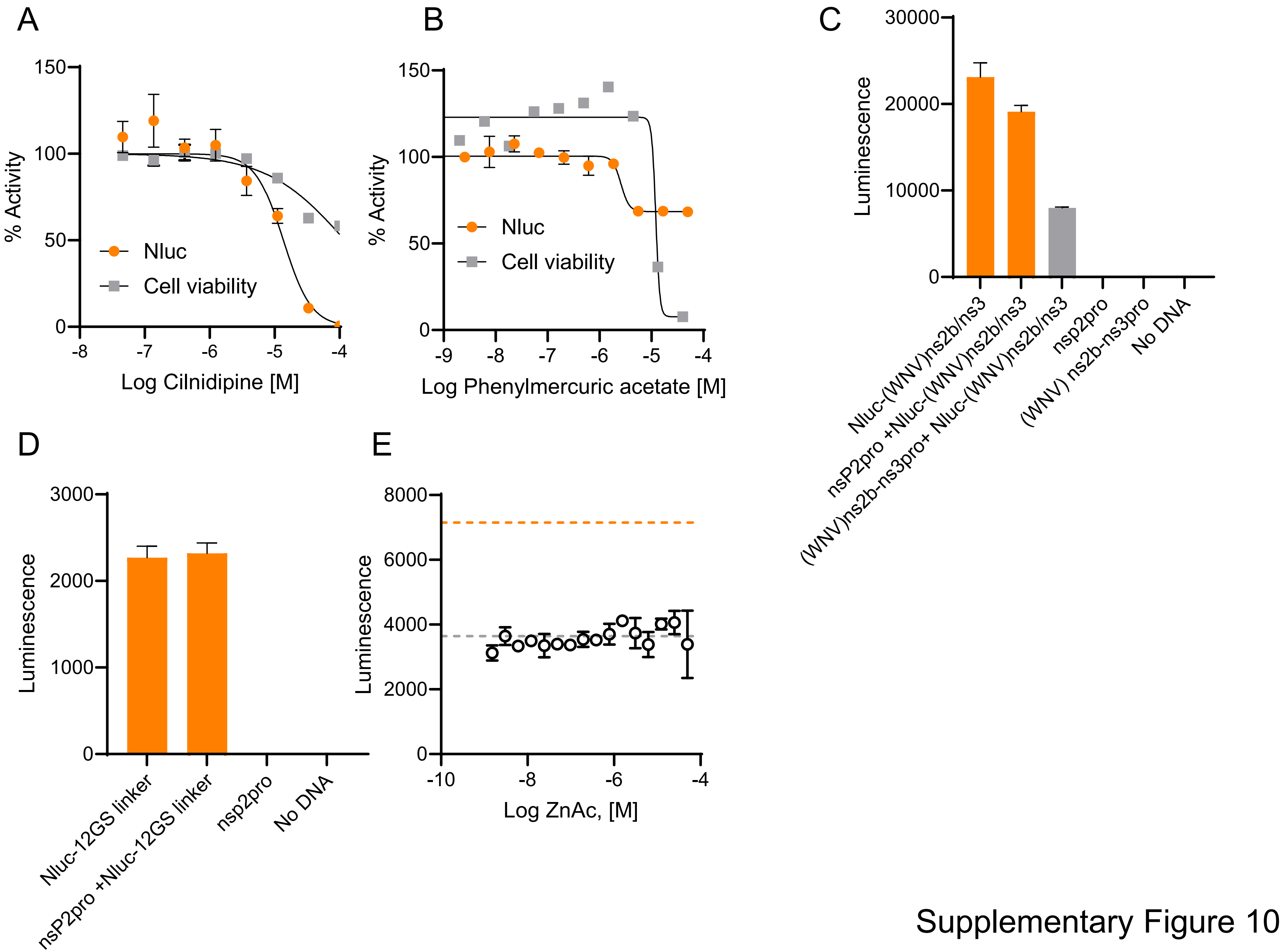

Supplementary Figure 10

F

				Cell-based		Enzymatic	
NCGC ID	Sample name	Library	Note	AC_{50} (µM)	% Efficacy	IC_{50} (µM)	% Efficacy
NCGC00098557		diversity		12.9	101.3	7.9	-80.1
NCGC00116366		diversity		36.5	309	4.5	-114.1
NCGC00116374		diversity		14.5	112.1	4	-102.7
NCGC00137618		diversity		32.6	55.5	10	-83
NCGC00241881		diversity	not selective	>50	179	1.1	-106.1
NCGC00400417		diversity	not selective	47.2	75.7	2.1	-107.3
NCGC00031503		repurposing		0.9	181.6	1.9	-118.4
NCGC00182060	l-Oxyfedrine	repurposing		2.6	60.3	1.3	-88.6
NCGC00386531	B-HT 958	repurposing		0.23	113.8	7.9	-62.1
NCGC00387487	SPP 86	repurposing		18.3	146.3	8	-114.8
NCGC00016542	Levonordefrin	repurposing	not selective	0.4	71.5	7.7	-74.9
NCGC00346545	Telaprevir	repurposing	not selective	31.6	212.6	3.5	-123.2
NCGC00487056	Semapimod	repurposing	not selective	2.2	136.8	6.3	-105.6

G

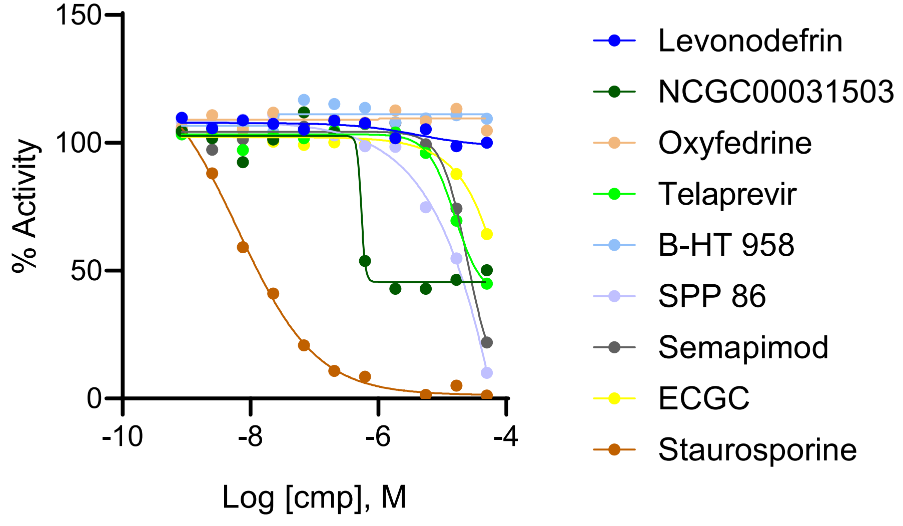

Supplementary Figure 10. Optimization of cell-based proteolytic assay. (A-B) Dose response curves of the Nluc inhibitor Cilnidipine (A) and the cytotoxic compound Phenylmercuric acetate (B) in the Nluc cell-based proteolytic assay (Nluc) and a CellTiter-Glo viability assays. (C) Cleavage of an Nluc reporter containing the West Nile Virus ns2b-ns3 cleavage site by WNV ns2b-ns3 protease but not CHKV nsP2pro. Data represents average +/- standard deviation of n=3. (D) nsP2pro does not cleave an Nluc reporter containing just a flexible 12X SerGly (12SG) loop. Data represents average +/- standard deviation of n=3. (E) ZnAc does not inhibit proteolytic cleavage of Nluc-nsp3/4 by nsp2pro in cell assays. Dotted lines indicate average luminescence values of control wells transfected with Nluc-nsp3/4 (orange) or Nluc-nsp3/4 and nsP2pro (grey). Data represents average +/- standard deviation of n=4. (F) AC50, IC50 and % Efficacy of 13 hits in cell-based and nsP2pro biochemical assay. (G) CellTiter-Glo viability assay of SNB19 cells treated with nsP2pro hits from repurposing libraries for 48hr. Data is normalized to DMSO (100%) and Staurosporine (0%).

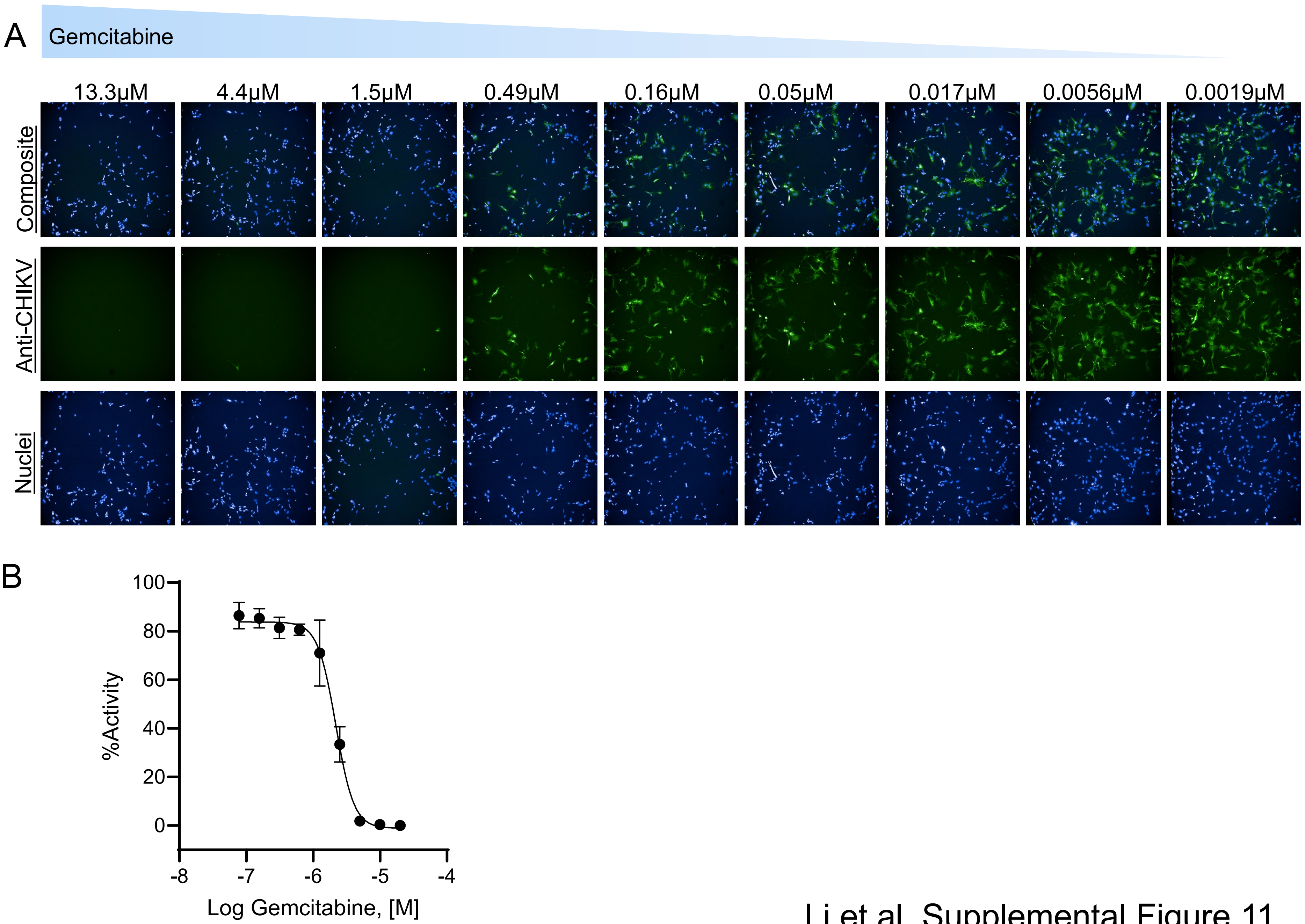

Li et al. Supplemental Figure 11

Supplementary Figure 11. Gemcitabine control in CHIKV viral assay. (A) Representative immunofluorescence images of CHIKV infection inhibited by a dose treatment of Gemcitabine in SNB-19 cells. Cell nuclei are stained with Hoechst 33342 (blue channel) and virus antigen detected with anti-CHIKV antibody recognizing CHIKV E1 protein (green channel). (B) Dose response curve for infection inhibition by Gemcitabine. Data represents average +/- standard deviation (n=3).

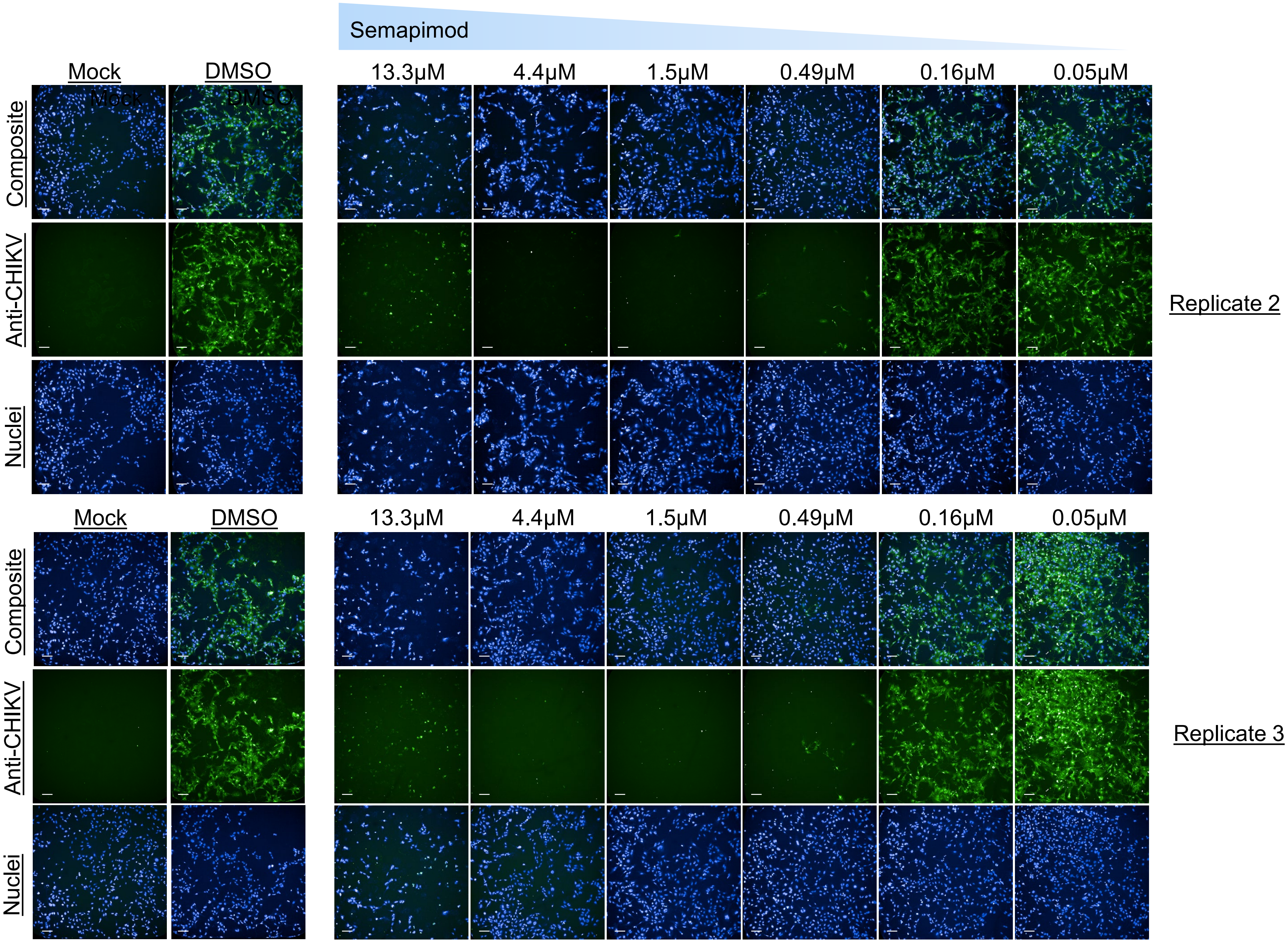

Li et al. Supplemental Figure 12

Supplementary Figure 12. CHIKV viral assay. (A) Representative immunofluorescence images of two additional replicates of CHIKV infection inhibited by a dose treatment of Semapimod in SNB-19 cells. Cell nuclei are stained with Hoechst 33342 (blue channel) and virus antigen detected with anti-CHIKV antibody recognizing CHIKV E1 protein (green channel).

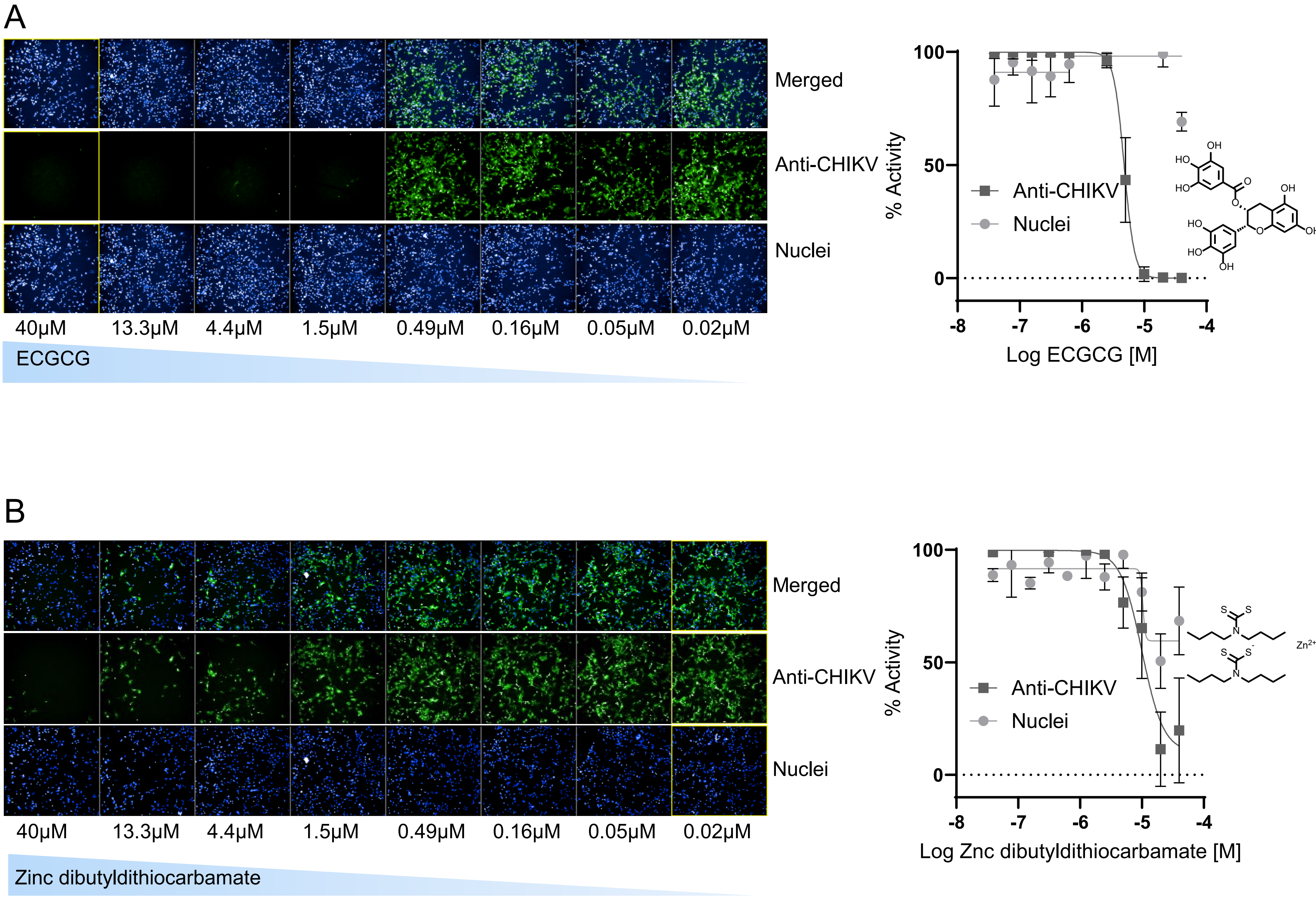

Supplementary Figure 13

Supplementary Figure 13. CHIKV viral assay. Left, representative immunofluorescence images of CHIKV inhibition by (A) EGCG and (B) Zn dibutyldithiocarbamate. Cell nuclei are stained with Hoechst 33342 (blue channel) and virus antigen detected with anti-CHIKV antibody recognizing CHIKV E1 protein (green channel). Right, dose response curve for infection and nuclei count. Data represents average +/- standard deviation (n=3).

Assay	Screen Type	Library	# Cmps	# Conc	S/B	Z'
nsp2pro+peptide 2	Pilot	Anti-infectives	739	7	3.92 ± 0.29	0.81 ± 0.05
		NPIC	9937		2.66 ± 0.11	0.71 ± 0.29
		NPC	2807		10.1 ± 1.2	0.83 ± 0.04
		NPACT	5448		5.11 ± 1.67	0.75 ± 0.09
	Diversity	miniSytravon	9365	5	5.67 ± 1.66	0.85 ± 0.03
		miniGenesis	9898	6	3.99 ± 1.57	0.77 ± 0.08
		extraGenesis	994	7	5.15 ± 0.178	0.86 ± 0.03
		extraSytravon	401	11	2.79 ± 0.26	0.70 ± 0.08
		NCGC Chem	8285	6	13.32 ± 1.63	0.78 ± 0.19
nsp2pro+peptide 2	Pilot	follow-up	251	11	5.31 ± 0.11	0.77 ± 0.04
Papain					5.50 ± 0.23	0.88 ± 0.01
HCV NS3-4A					2.55 ± 0.05	0.17 ± 0.01
hFurin					17.5 ± 1.9	0.88 ± 0.02
nsP2pro+peptide 3					2.67 ± 0.18	0.86 ± 0.01
nsP2 FL+peptide 2					5.21 ± 0.06	0.77 ± 0.01
cell-based nsP2					3.99± 0.1	0.37 ± 0.08
nsp2pro+peptide 2	Diversity	follow-up	166	11	16.2 ± 0.83	0.89 ± 0.05
Papain					8.96 ± 0.74	0.88 ± 0.01
HCV NS3-4A					4.05 ± 0.11	0.72 ± 0.05
hFurin					24.0 ± 4.6	0.89 ± 0.02
nsP2pro+peptide 3					4.00 ± 0.25	0.86 ± 0.03
nsP2 FL+peptide 2					4.06 ± 0.2	0.58 ± 0.05
cell-based nsP2					2.2± 0.40	0.12 ± 0.14

Supplementary Figure 14. Assay statistics. Signal-to-background (S/B) and Z-scores for each assay. Library name, number of compounds tested, and number of concentrations tested are also indicated.
